# DA-DRD5 signaling controls colitis by regulating colonic M1/M2 macrophage polarization

**DOI:** 10.1038/s41419-021-03778-6

**Published:** 2021-05-17

**Authors:** Lu Liu, Yuqing Wu, Bingwei Wang, Yuying Jiang, Lin Lin, Xiaoxi Li, Shuo Yang

**Affiliations:** 1 Department of Immunology, Key Laboratory of Immunological Environment and Disease, State Key Laboratory of Reproductive Medicine, Center for Global Health, Nanjing Medical University, Nanjing, 211166 China; 2 Department of Pharmacology, Nanjing University of Chinese Medicine, Nanjing, China; 3 Department of Gastroenterology, The First Affiliated Hospital of Nanjing Medical University, Nanjing, China

**Keywords:** Inflammation, Monocytes and macrophages

## Abstract

The decrease of neurotransmitter dopamine (DA) levels in the intestine is closely related to the development of inflammatory bowel disease (IBD). However, the functional relevance and underlying mechanistic basis of the effects of DA signaling on IBD remains unclear. Here, we observed that the DRD5 receptor is highly expressed in colonic macrophages, and the deficiency of DA-DRD5 signaling exacerbated experimental colitis. Moreover, DA-DRD5 signaling can inhibit M1 by negatively regulating NF-κB signaling but promote M2 macrophage polarization through activation of the CREB pathway, respectively. The deficiency of DRD5 signaling increased colonic M1 macrophages but reduced M2 cells during colitis. Additionally, the administration of a D1-like agonist that has a higher affinity to DRD5 can attenuate the colitogenic phenotype of mice. Collectively, these findings provide the first demonstration of DA-DRD5 signaling in colonic macrophages controlling the development of colitis by regulating M1/M2 macrophage polarization.

## Introduction

Inflammatory bowel disease (IBD) is a group of chronic relapsing inflammatory conditions of the colon and small intestine mainly comprising Crohn’s disease (CD) and ulcerative colitis (UC)^1^. As the most sophisticated immune organ of the entire body, the extensive repertoire of intestinal immune cells is unique and plays an important role in IBD development. In particular, large numbers of macrophages are present in the intestine and the shift on macrophage phenotype has been implicated in the establishment of IBD^2,3^. M1 macrophages are classic inflammatory cells secreting proinflammatory cytokines and directly contribute to the defect of the barrier in IBD^4^. It was shown that in DSS-induced colitis mice, the population of M1 macrophages increases^5^. Conversely, M2 macrophages express anti-inflammatory cytokines, including IL-10, and are involved in tissue repair and inflammation resolution to relieve IBD^6^. Transfer of properly polarized M2 macrophages to *STAT6*^*−/−*^ mice accelerated wound healing in the damaged mucosa^7^. Thus, the regulation of M1/M2 macrophages balance might be a potential therapeutic strategy for IBD.

Additionally, it is becoming increasingly clear that normal gastrointestinal (GI) function depends on not only immune-cell populations but also the highly coordinated responses of the enteric nervous system (ENS)^8^. The neurons of the enteric nervous system control the functions of the GI system and communicate through various neurotransmitters, including dopamine (DA), acetylcholine, and serotonin, etc^9^. In the inflamed mucosa of CD and UC patients, the DA levels were markedly lower than in controls^10^. The study has shown that DA protects the homeostasis of the GI mucosal barrier through regulation of mucus secretion, intracellular pH, and submucosal blood flow^11^. In addition to its effects on gastrointestinal secretory responses, DA also acts as an inhibitory modulator of gastrointestinal motility^12^. Although these studies have established a framework of the relationship between DA and IBD, the functions and their underlying mechanisms of DA in the regulation of mucosal immunity and colitis remain not well understood.

To date, DA has been reported as an essential regulator of different immune cells by acting on its two primary subfamilies receptors: D1-like dopamine receptors that comprise dopamine receptor D1 (DRD1) and dopamine receptor D5 (DRD5); D2-like dopamine receptors, including dopamine receptor D2 (DRD2), dopamine receptor D3 (DRD3) and dopamine receptor D4 (DRD4)^13^. There has been some progress in understanding the role of DA receptors in regulating immune cell functions. Follicular helper T (TFH) cell-derived DA enhances T-B cell interactions through the DRD1 of germinal center B cells^14^. DRD2 activation in astrocytes can suppress neuroinflammation and the development of Parkinson’s disease in the central nervous system via αB-crystallin protein^15^. Dopaminergic agonists can reduce the ovalbumin antigen-induced activation of neutrophils via D1-like receptors in a mouse model of airway inflammation^16^. DA inhibits NLRP3 inflammasome activation and system inflammation via DRD1 signaling in macrophages^17^. More recently, we described the detailed role and mechanisms of the macrophage DA-DRD5 signaling in controlling inflammation-associated diseases such as meningitis and sepsis^18^. Thus, these studies highlight DA has an important influence on immune cell functions. However, the underlying functions of DA in modulating intestinal immune cells are largely unknown.

In this study, we found that DRD5 is highly expressed in colonic macrophages and DRD5 deficiency exacerbates DSS-induced colitis. Moreover, DRD5 signaling rebalances the colonic M1/M2 macrophage ratio by negatively regulating NF-κB signaling and activating of the CREB pathway, and then the administration of DRD5 agonist attenuates the colitogenic phenotype of mice. Thus, we proposed a novel neuroimmune regulatory pathway in which DA corrects the M1/M2 excessive polarization through DRD5 to alleviate intestinal inflammation.

## Results

### DRD5 receptor is highly expressed in colonic macrophages

**Fig. 1:**
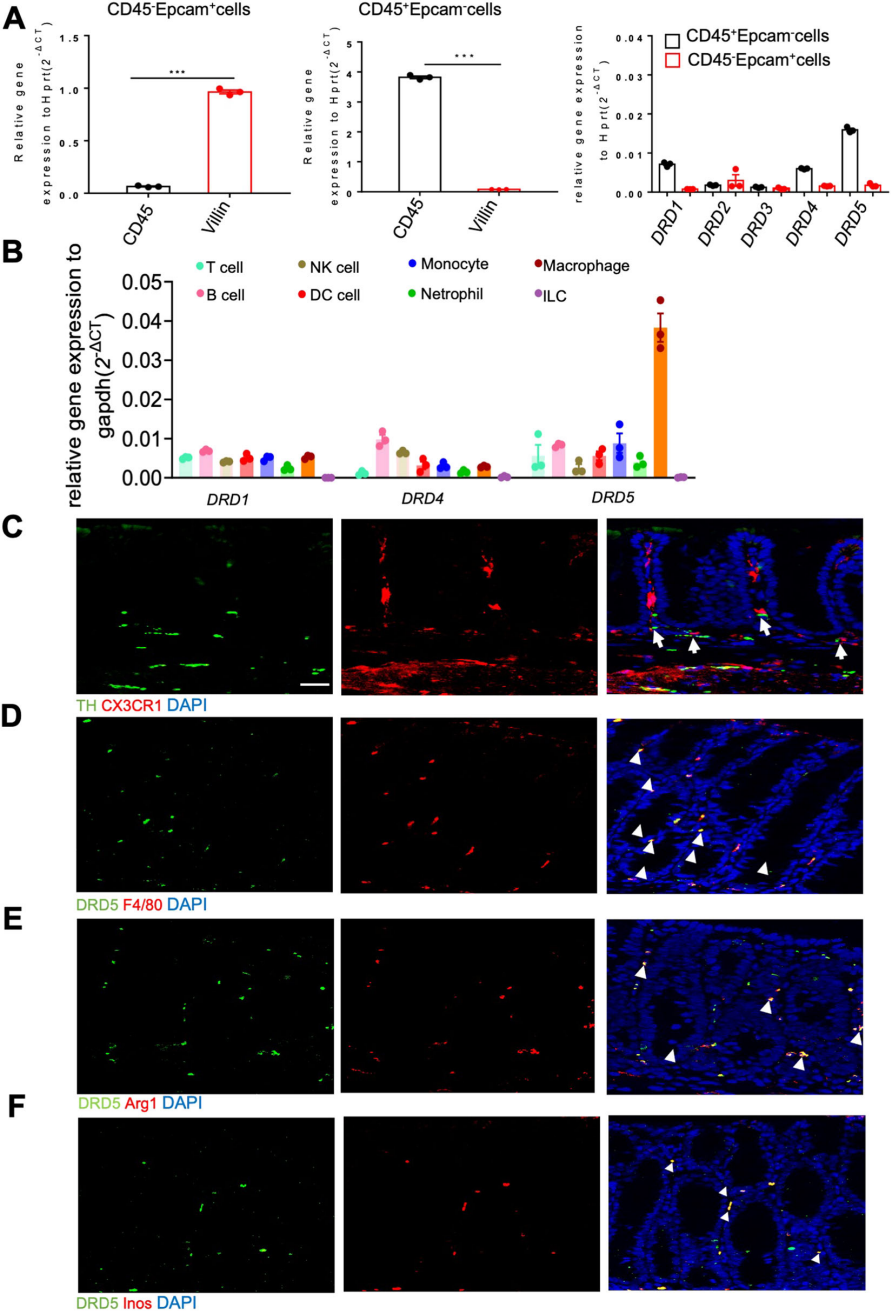
DRD5 receptor is highly expressed in colonic macrophages. **A** RT-qPCR analysis of gene expression of *DRD1*, *DRD2*, *DRD3*, *DRD4*, and *DRD5* in IECs (CD45^-^Epcam^+^) and LP cells (CD45^+^Epcam^-^) isolated from WT mice (*n* = 3 mice per group). **B** RT-qPCR analysis of gene expression of *DRD1*, *DRD4*, and *DRD5* in T cells, B cells, NK cells, DC cells, monocytes, neutrophils, macrophages, and ILCs cells isolated from the colonic LP of WT mice (*n* = 3 mice per group). **C** Immunofluorescent labeling of TH (green), Cx3cr1(red), and DAPI (blue) in colon sections from Cx3cr1 reporter mice. The close proximity of macrophages with dopaminergic neurons is indicated by arrow. Scale bar, 30 µm. **D** Immunofluorescent labeling of DRD5 (green), F4/80 (red), and DAPI (blue) in colon sections from WT mice. The merging of DRD5 with F4/80^+^ in macrophages indicated by the arrowhead. Scale bar, 30 µm. **E** Immunofluorescent labeling of DRD5 (green), Arg1(red), and DAPI (blue) in colon sections from WT mice. The merging of DRD5 with Arg1^+^ in macrophages indicated by the arrowhead. Scale bar, 30 µm. **F** Immunofluorescent labeling of DRD5 (green), Inos (red), and DAPI (blue) in colon sections from WT mice. The merging of DRD5 with Inos^+^ in macrophages indicated by the arrowhead. Scale bar, 30 µm. Data are pooled from three independent experiments (**A**, **B**). Error bars show means ± SEM. ***p < 0.001. Two-tailed unpaired student’s *t*-test.

To systematically investigate the roles of DA-DRDs signaling in the gut mucosal immune system and colitis, we firstly tested the expression of DA receptors in the isolated epithelial and CD45^+^ lamina propria (LP) hematopoietic cells (Fig. S1A) from the colon. We found that DRD1, DRD4, and DRD5 were highly expressed in CD45^+^LP cells (Fig. 1A), whereas the expression of DRD2 and DRD3 were very low in both epithelial and LP immune cells. Since LP contains a range of immune cells that maintain homeostasis and respond to a breakdown of epithelial protection^19^, we further measured the expression of DRD1, DRD4 and DRD5 in various immune cells of LP, including Macrophages (CD45^+^CD11b^+^F4/80^+^), Monocytes (CD45^+^CD11b^+^Ly6c^+^), NK cells (CD45^+^ NK1.1^+^), Neutrophils (CD45^+^Ly6g^+^), Dendritic cells (CD45^+^ CD11c^+^), T cells (CD45^+^ TCR-β^+^), B cells (CD45^+^ CD19^+^), ILCs (CD45^+^ Lin^-^) (Fig. S1B). Notably, a significantly high expression of DRD5 in macrophages was observed by RT-PCR analysis (Fig. 1B). Immunofluorescence analysis showed that CX3CR1^+^ macrophages were close to tyrosine hydroxylase (TH) positive neurons in LP layer (Fig. 1C). Besides, we observed the wide distribution of DRD5 signaling in LP F4/80^+^ macrophages (Fig. 1D), Arg1^+^M2 macrophages, and Inos^+^M1 macrophages (Fig. 1E, F). Thus, these data suggest that DA-DRD5 signaling could have an important regulatory effect on colonic macrophage function.

### DRD5 deficiency in immune cells exacerbates DSS-induced colitis

**Fig. 2:**
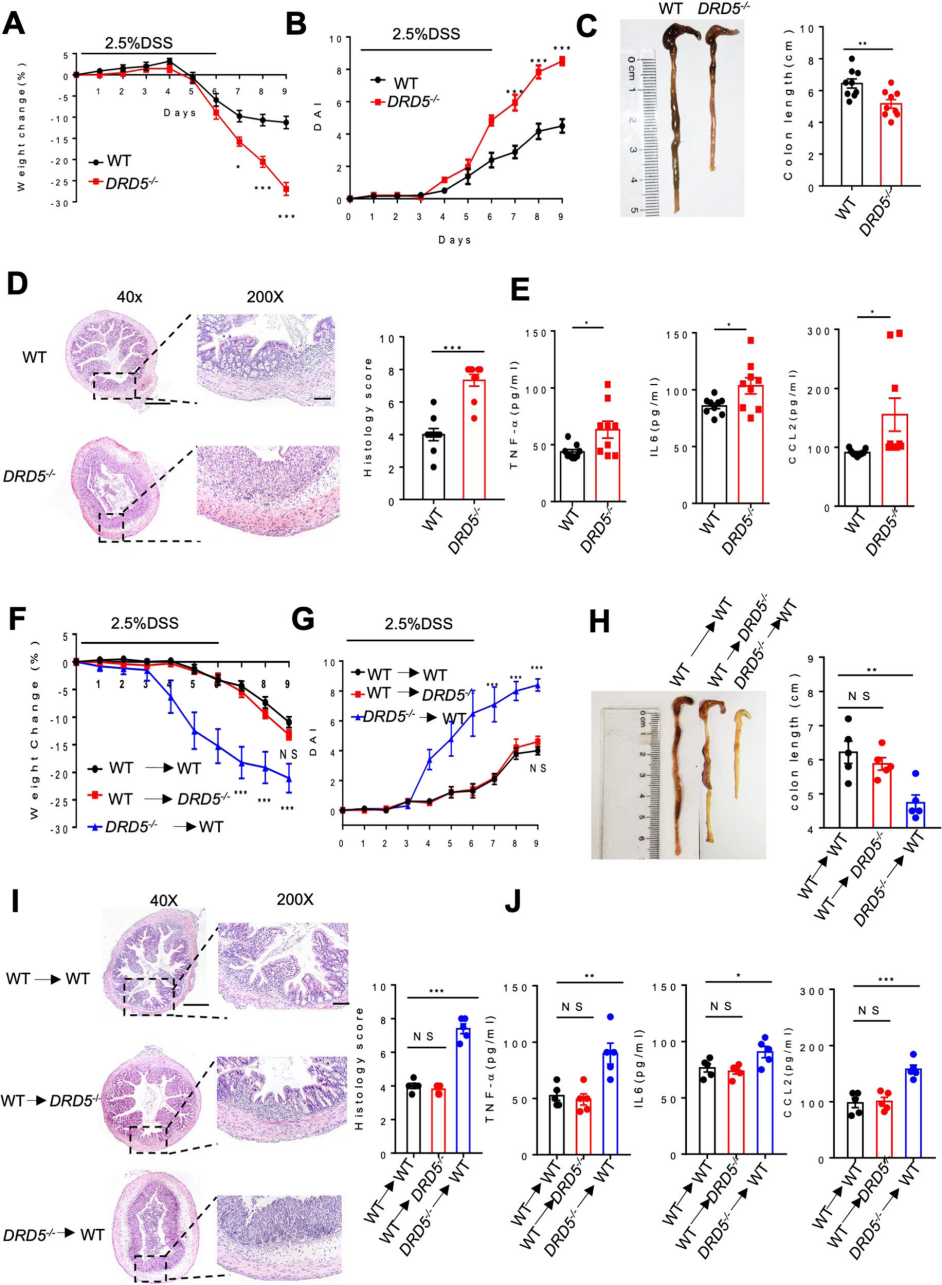
DRD5 deficiency in immune cells exacerbates DSS-induced colitis. **A**, **B** Age-matched male WT and *DRD5*^−*/−*^ mice (*n* = 9 mice per group) were given 2.5% DSS in their drinking water for 6 days and distilled water for three additional days before sacrifice. Weight changes (**A**) and disease activity index (DAI) (**B**) were monitored daily. **C** Gross morphology images of colons and colon lengths of WT and *DRD5*^−*/*−^ mice on day 9 after DSS treatment. **D** Representative H&E-stained colonic sections and histology scores of WT and *DRD5*^−*/*−^ mice sampled on day 9 after DSS treatment. Scale bars, 4×, 500 μm; 20×, 200 μm. **E** The concentration of TNF-α, IL-6, and CCL2 in the serum of WT and *DRD5*^−*/−*^ mice were measured by ELISA. **F**, **G** Weight changes (**F**) and disease activity index (DAI) (**G**) after DSS-induced colitis in WT and *DRD5*^−*/*−^ mice (*n* = 5 mice per group) adoptively transferred with WT or *DRD5*^*−/−*^ bone marrow cells. **H** Gross morphology images of colons and colon lengths of WT and *DRD5*^−*/−*^ mice adoptively transferred with WT or *DRD5*^−*/*−^ bone marrow cells on day 9 after DSS treatment. **I** Representative H&E staining of colonic sections and histology scores of WT and *DRD5*^−*/−*^ mice adoptively transferred with WT or *DRD5*^−*/−*^ bone marrow cells on day 9 after DSS treatment. Scale bars, 4×, 500 μm; 20×, 200 μm. **J** The concentration of TNF-α, IL-6, and CCL2 in the serum of WT and *DRD5*^*−/*−^ mice (*n* = 5 mice per group) adoptively transferred with WT or *DRD5*^−*/−*^ bone marrow cells after DSS treatment. Data are pooled from three independent experiments. Error bars show means ± SEM. *P < 0.05; **P < 0.01; ***P < 0.001. multiple unpaired *t*-tests for (**A**, **B**, **F**, **G**) and two-tailed unpaired student’s *t*-test for (**C**–**E**). One-way ANOVA with Sidak’s multiple comparisons test for (**H**–**J**).

Notably, the public data sets (GSE47908 and GSE38713) showed that the gene expression of DRD5 was significantly decreased in the colons of UC patients or active UC patients (Fig. S2A). To evaluate the role of DRD5 signaling in colitis, we used the dextran sodium sulfate (DSS) model of colitis in mice. Briefly, mice were given 2.5% DSS in the drinking water for six days and then switched to regular drinking water until day 9. First, we observed the colonic DA levels were markedly reduced after DSS treatment from 1.5 × 10^−7^ M to 1 × 10^−7^ M (Fig. S2B), further suggesting the close relation of decrease of DA levels with colitis. Next, we treated age-matched DRD5 knockout (*DRD5*^*−/−*^) or wild-type (WT) mice with DSS to compare their colitis phenotype. Although no obvious differences in the weight, histopathology of colons between WT and *DRD5*^−/−^ mice were observed before DSS treatment (Fig. S2C, D), more severe colitis on day nine after DSS administration was observed in *DRD5*^*−/−*^ mice than in WT controls, as characterized by significantly greater body weight loss, higher disease activity index (DAI) score, and shorter colons in DSS-treated *DRD5*^*−/−*^ mice (Fig. 2A–C). Histopathological analysis [hematoxylin and eosin (H&E)] further showed that DRD5 deficiency increased inflammatory cell infiltration with more damage of mucosal epithelium (Fig. 2D). Also, the serum levels of TNF-α, IL-6, and CCL2 were markedly increased in the *DRD5*^*−/−*^ group compared with the WT group (Fig. 2E). Previous studies have suggested that the dynamic shifts in gut microbiota play a role during the development of colitis^20^. To evaluate whether microbiota variation was associated with the increased severity in colitis observed in *DRD5*^*−/−*^ mice, we cohoused littermate WT mice [WT (*DRD5*^*−/−*^)] and *DRD5*^*−/−*^ mice [*DRD5*^*−/−*^ (WT)] for 6 weeks to roughly equalize bacterial community before the administration of DSS. The 16s rRNA Sequencing showed that the microbial community structure and composition of single-housed *DRD5*^*−/−*^ mice were different from WT group, but they tend to be consistent after cohousing (Fig. S3A, B). Moreover, *DRD5*^*−/−*^ single-housed mice and cohoused mice had slightly high abundance of colitis-associated microbiota, such as Prevotellaceae and Clostridia_UCG-014, and low abundance of protective bacteria, including Bacteroidaceae and Tannerellaceae, which indicating *DRD5*^*−/−*^ mice have a weak tendency to develop colitis (Fig. S3C). However, cohousing breeding did not change the development of more severe DSS-induced colitis in *DRD5*^*−/−*^ (WT) mice compared with the cohoused WT (*DRD5*^*−/−*^) controls (Fig. S3D–G), suggesting microbiota changes did not involve in the protection of DRD5 against colitis directly.

To further determine whether DRD5 deficiency in immune cells or gut-resident cells contributes to the increased severity of colitis, we firstly generated bone marrow chimeric mice by adoptively transferring WT or *DRD5*^*−/−*^ bone marrow cells into lethally irradiated WT recipient mice. We found that WT mice reconstituted with *DRD5*^−/−^ bone marrow was easier to induce colitis with greater body weight loss, higher DAI score, and shorter colons than WT donors after DSS administration (Fig. 2F–H). H&E staining showed the more infiltrating inflammatory cells and more severe disruption in the colon of *DRD5*^*−/−*^ donor mice relative to WT donors during colitis (Fig. 2I). The levels of TNF-α, IL-6, and CCL2 were also increased in *DRD5*^*−/−*^ donor mice compared with the WT donor mice (Fig. 2J). However, a reverse bone marrow transfer experiment in which lethally irradiated WT or *DRD5*^*−/−*^ mice recipient mice were reconstituted with bone marrow cells isolated from WT, demonstrated the comparable body weight loss, DAI scores, colon length, colon pathology, and serum inflammatory cytokines between these two recipients (Fig. 2F–J). Therefore, these results suggest that DRD5 signaling in immune cells is essential for its protective role in DSS-induced colitis.

### The deficiency of DA-DRD5 signaling inhibits M1 but enhances M2 macrophage polarization in vitro

**Fig. 3:**
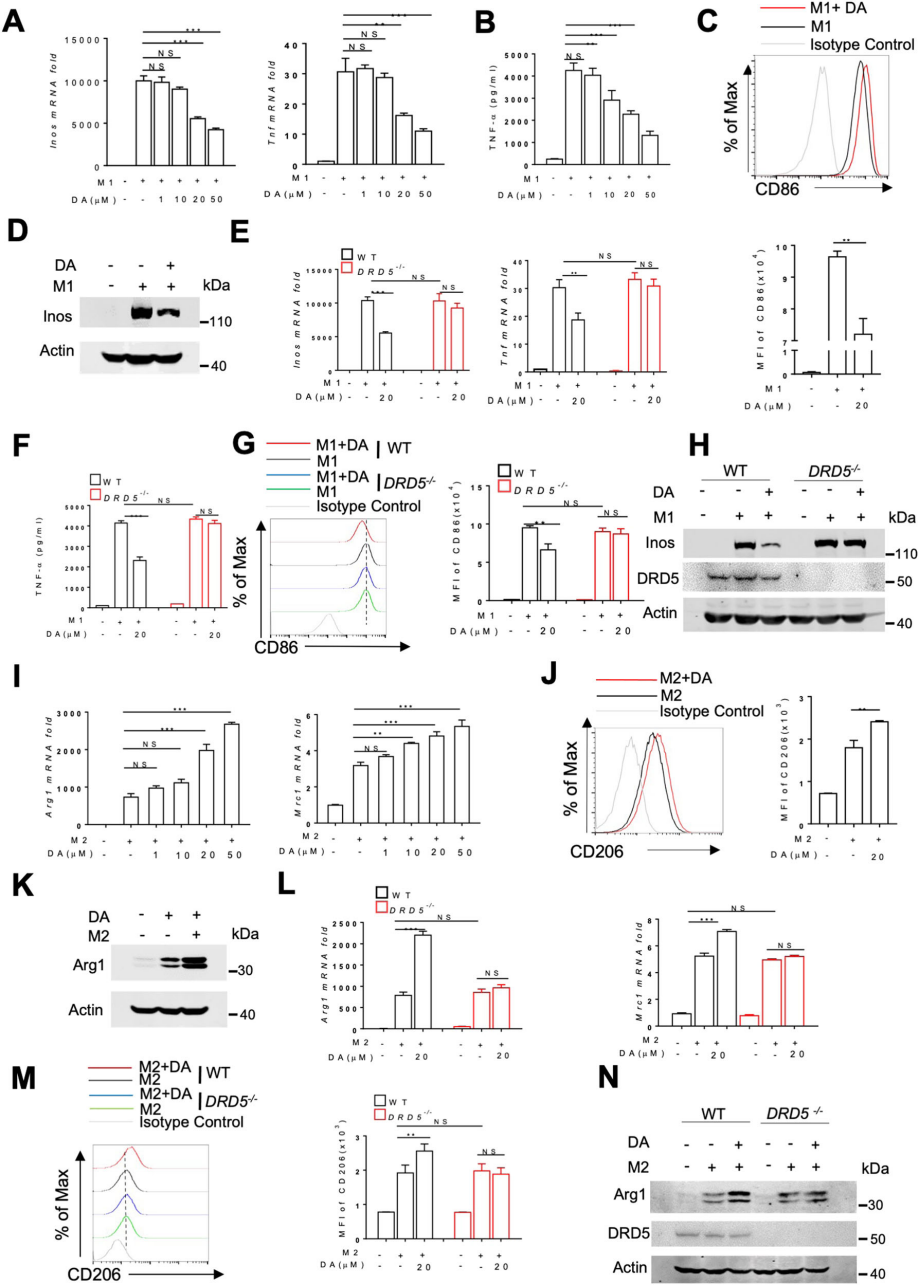
The deficiency of DA-DRD5 signaling inhibits M1 but enhances M2 macrophage polarization in vitro. **A** RT-qPCR analysis of *Inos* and *Tnf* mRNA expression in WT BMDMs treated with various doses of DA and stimulated with LPS/IFN-γ (M1) for 12 h. **B** ELISA analysis of TNF-α in supernatants from WT BMDMs treated with various doses of DA and stimulated with LPS/IFN-γ (M1) for 12 h. **C** Flow cytometry analysis of M1 marker CD86 of WT BMDMs treated with DA (20 μM) and stimulated with LPS/IFN-γ (M1) for 12 h. Representative overlaid flow cytometry histograms showing mean fluorescence intensity (MFI) (top) and quantified data from three independent experiments (*n* = 3 per group) (bottom). **D** Immunoblot analysis of Inos in lysates of WT BMDMs treated with DA (20 μM) and stimulated with LPS/IFN-γ (M1) for 12 h. **E** RT-qPCR analysis of *Inos* and *Tnf* mRNA expression in WT and *DRD5*^*−/−*^ BMDMs treated with DA (20 μM) and stimulated with LPS/IFN-γ (M1) for 12 h. **F** ELISA analysis of TNF-α in supernatants from WT and *DRD5*^*−/−*^ BMDMs treated with DA (20 μM) and stimulated with LPS/IFN-γ (M1) for 12 h. **G** Flow cytometry analysis of M1 marker CD86 of WT and *DRD5*^*−/−*^ BMDMs treated with DA (20μM) and stimulated with LPS/IFN-γ (M1) for 12 h. Representative overlaid flow cytometry histograms showing mean fluorescence intensity (MFI) (left) and quantified data from three independent experiments (*n* = 3 per group) (right). **H** Immunoblot analysis of Inos in lysates of WT and *DRD5*^*−/−*^ BMDMs treated with DA (20 μM) and stimulated with LPS/IFN-γ (M1) for 12 h. **I** RT-qPCR analysis of *Arg1* and *Mrc1* mRNA expression in WT BMDMs treated with various doses of DA and stimulated with IL-4/IL-13 (M2) for 12 h. **J** Flow cytometry analysis of M2 marker CD206 of WT BMDMs treated with DA (20 μM) and stimulated with IL-4/IL-13 (M2) for 12 h. Representative overlaid flow cytometry histograms showing mean fluorescence intensity (MFI) (left) and quantified data from three independent experiments (*n* = 3 per group) (right). **K** Immunoblot analysis of Arg1 in lysates of WT BMDMs treated with DA (20 μM) and stimulated with M2 for 12 h. **L** RT-qPCR analysis of *Arg1* and *Mrc1* mRNA expression in WT and *DRD5*^*−/−*^ BMDMs treated with DA (20 μM) and stimulated with IL-4/IL-13 (M2) for 12 h. **M** Flow cytometry analysis of M2 marker CD206 of WT and *DRD5*^*−/−*^ BMDMs treated with DA (20 μM) and stimulated with IL-4/IL-13 (M2) for 12 h. Representative overlaid flow cytometry histograms showing mean fluorescence intensity (MFI) (left) and quantified data from three independent experiments (*n* = 3 per group) (right). **N** Immunoblot analysis of Arg1 in lysates of WT and *DRD5*^*−/−*^ BMDMs treated with DA (20 μM) and stimulated with IL-4/IL-13 (M2) for 12 h. Data are pooled from three independent experiments. Error bars show means ± SEM. *p < 0.05, **p < 0.01, ***p < 0.001; NS, not significant. One-way ANOVA with Sidak’s multiple comparisons test for (**A**–**C, E**–**G, I**–**J, L**–**M**).

Previous studies by us and other research groups have shown that DA can inhibit inflammation in macrophages^17,18^. Given M1/M2 macrophages present in the intestine play essential roles in the initiation or resolution of inflammation in IBD^21^, we speculate DA may have an important role in the polarization of macrophages. To test this, we first used Lps and Ifn-γ to treat BMDMs in vitro for M1 macrophage differentiation in the presence or absence of DA. We found that DA treatment significantly inhibited Lps/Ifn-γ-induced expression of M1-associated genes, including *Inos*, *Tnf*, and *Il6* at the mRNA level in a dose-dependent manner (Fig. 3A and Fig. S4A). Meanwhile, ELISA analysis showed DA decreased the protein levels of TNF-α and IL-6 in a dose-dependent way (Fig. 3B and Fig. S4A). Consistent with previous reports showing that DA is subject to degradation by monoamine oxidase (MAO) and catechol-O-methyltransferase (COMT) to shorten its half-life^22^, we found that co-treatment with MAO and COMT inhibitors can greatly improve the inhibitory effect of DA at the DA concentration of 0.5 μM (Fig. S4B, C). Moreover, fluorescence-activated cell sorting (FACS) revealed that DA markedly reduced the expression of CD86 on macrophages under M1 conditions (Fig. 3C). The immunoblotting analysis also demonstrated DA significantly reduced the expression of Inos protein after Lps/Ifn-γ treatment (Fig. 3D). Thus, these data suggest an inhibitory role of DA in M1 macrophage polarization.

Given that DRD5 is highly expressed in colonic macrophages, we next address whether DA regulates M1 macrophage polarization through DRD5. Notably, the inhibitory effect of DA on the mRNA expression levels of M1-associated genes, including *Inos*, *Tnf*, and *Il6* in response to Lps/Ifn-γ was completely lost in *DRD5*^*−/−*^ BMDMs (Fig. 3E and Fig. S4F). ELISA analysis showed that such inhibitory effects of DA on TNF-α and IL-6 production in response to Lps and Ifn-γ stimulation were severely impaired in *DRD5*^*−/−*^ cells (Fig. 3F and Fig. S4F). Also, FACS revealed *DRD5*^*−/−*^ cells were resistant to the inhibitory effects of DA on CD86 expression under M1 conditions (Fig. 3G). Moreover, Immunoblotting analysis demonstrated DRD5 deficiency precluded the inhibitory effect of DA on the expression of Inos protein in macrophages (Fig. 3H). Altogether, these data suggest that DA-DRD5 signaling inhibits M1 macrophage polarization.

Next, we assessed whether DA regulates M2 macrophage differentiation in response to IL-4 and IL-13 stimulation. Interestingly, we observed that DA markedly augmented the expression of M2-associated genes, including *Arg1*, *Mrc1*, and *Ym1*, in response to the IL-4/IL-13 treatment (Fig. 3I and Fig. S4D). Also, MAO and COMT inhibitors can improve the promotion effects of DA on M2 macrophages at the lowest concentration of 0.5 μM (Fig. S4E). FACS revealed that DA could increase the induction of CD206^+^ M2 macrophages in response to IL-4/IL-13 (Fig. 3J). Moreover, the significantly increased expression of Arg1 at the protein level was detected in M2 macrophages with DA treatment compared to the controls (Fig. 3K). In contrast to WT cells, DA failed to increase the expression of M2-associated genes, including *Arg1*, *Mrc1*, and *Ym1* in *DRD5*^*−/−*^ cells (Fig. 3L and Fig. S4G). Additionally, FACS revealed the increased CD206 expression on M2 macrophages in the presence of DA was severely impaired in *DRD5*^*−/−*^ cells (Fig. 3M). Moreover, the immunoblotting analysis demonstrated loss of DRD5 impaired the promoted effect of DA on the expression of Arg1 protein in macrophages (Fig. 3N). Thus, these results suggested that DA can also promote M2 macrophage polarization through DRD5.

### DA-DRD5 signaling can inhibit M1 macrophages polarization by negatively regulating NF-κB signaling, and promote M2 macrophages polarization through the activation of the CREB pathway

**Fig. 4:**
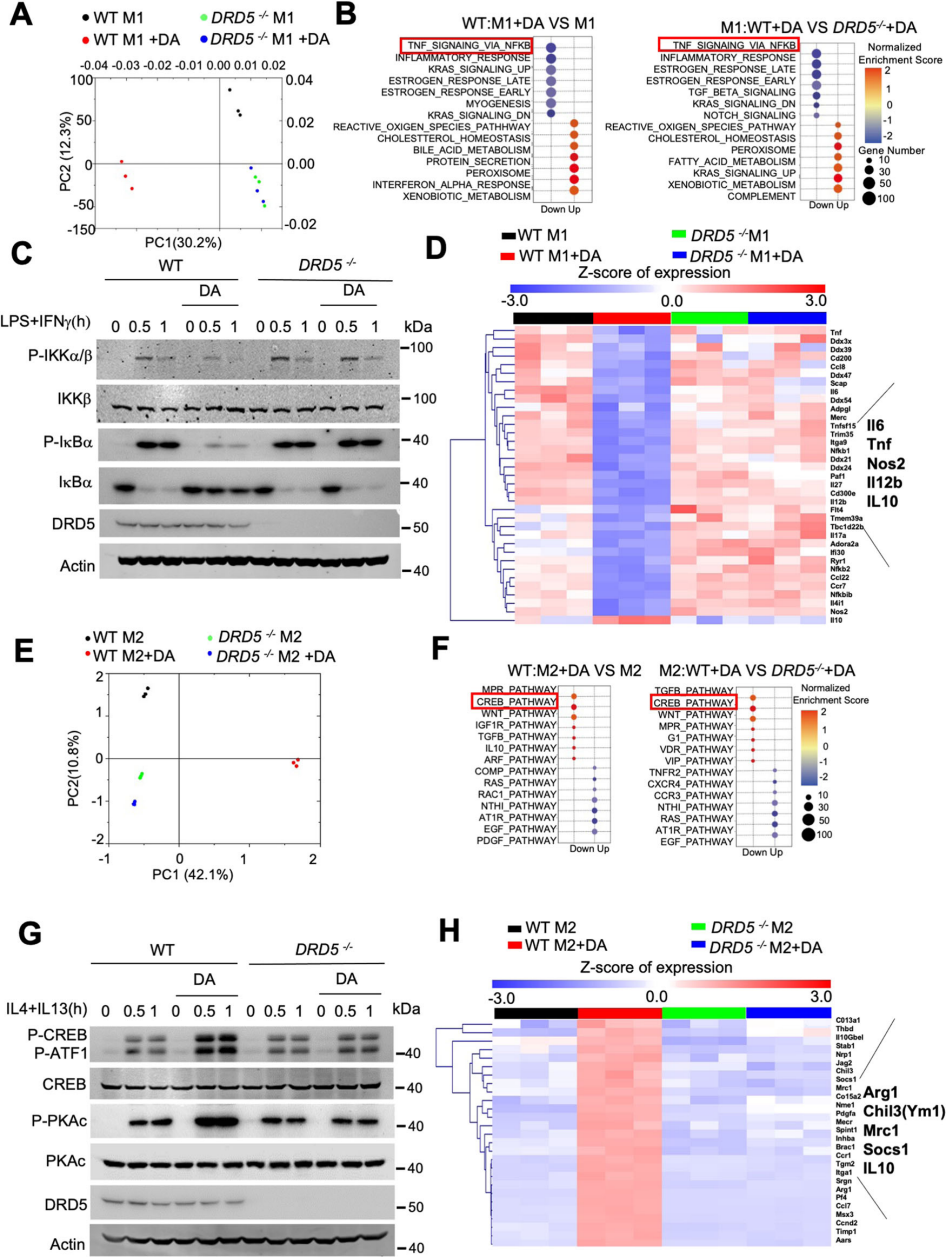
DA-DRD5 signaling can inhibit M1 polarization by negatively regulating NF-κB signaling and promote M2 polarization through the activation of the CREB pathway. **A** Principal component (PC) analysis of transcriptional clustered architecture in M1-stimulated WT and *DRD5*^*−/−*^ BMDMs untreated or treated with DA (20 μM) (*n* = 3). **B** Gene set enrichment analysis (GSEA) analysis of the prominent down-regulated pathways in M1-stimulated WT BMDMs untreated or treated with DA (left) or M1-stimulated WT and *DRD5*^*−/−*^ BMDMs treated with DA (right). **C** Immunoblot analysis of phosphorylated (p−), and total IKKα/β, IκBα in lysates of LPS/IFN-γ-stimulated WT and *DRD5*^*−/−*^ BMDMs untreated or treated with DA (20 μM) as indicated. **D** Heat map showing the expression of NF-κB-mediated M1 genes in LPS/IFN-γ-stimulated WT and *DRD5*^*−/−*^ BMDMs untreated or treated with DA. **E** PC analysis of gene expression cluster in M2-stimulated WT and *DRD5*^*−/−*^ BMDMs untreated or treated with DA (20 μM) (*n* = 3). **F** GSEA analysis of the prominent upregulated pathways in M2-stimulated WT BMDMs untreated or treated with DA (left) or M2-stimulated WT and *DRD5*^*−/−*^ BMDMs treated with DA (right). **G** Immunoblot analysis of phosphorylated (p−), and total CREB, PKAc in lysates of IL-4/IL-13-stimulated WT and *DRD5*^*−/−*^ BMDMs untreated or treated with DA (20 μM) as indicated. **H** Heat map showing the expression of genes associated with M2 macrophage in IL-4/IL-13-stimulated WT and *DRD5*^*−/−*^ BMDMs untreated or treated with DA. Data are representative of three independent experiments (**C**, **G**).

To further dissect the mechanistic of DA in regulating the balance of M1/M2 macrophage polarization, we performed RNA sequencing to analyze the transcriptional profiles of Lps/Ifn-γ-stimulated M1 WT and *DRD5*^*−/−*^ macrophage in the absence or presence of DA. Principal component (PC) analysis showed that the profiles of DA-treated M1 WT BMDMs had a distinct gene clustered architecture compared with the communities of M1 WT, M1 *DRD5*^*−/−*^, and DA-treated M1 *DRD5*^*−/−*^ cells (Fig. 4A). Gene set enrichment analysis (GSEA) revealed that the most prominent down-regulated pathways in DA-treated M1 WT BMDMs were the TNFA_SIGNALING_VIA_NFKB compared with untreated M1 WT cells. Moreover, *DRD5*^*−/−*^ BMDMs treated with DA were also enriched with the NFκB pathway relative to DA-treated WT cells (Fig. 4B). This finding is consistent with our previous report that dopamine uses the DRD5 signaling to block TRAF6-mediated NF-κB activation and inflammation^18^. Accordingly, we found that DA remarkably reduced the capacity of Lps /Ifn-γ to induce phosphorylation of IKKs or of their substrate IκBα, both of which are activation indices of the canonical NF-κB pathway. Moreover, IκBα degradation after stimulation with Lps/Ifn-γ was significantly impaired in the presence of DA. However, such inhibitory effect of DA on the phosphorylation of IKKs and the degradation of IκBα under M1 conditions was completely lost in BMDMs from *DRD5*^*−/−*^ lines (Fig. 4C). Additionally, the heat map showed that DA treatment inhibited the expression of NF-κB-mediated M1 genes, including *Il6*, *Tnf*, *Nos2*, *Il12b* in WT but not *DRD5*^*−/−*^ BMDMs after stimulation with Lps/Ifn-γ (Fig. 4D). Besides, consistent with the previous findings that DA/DRD5 plays anti-NF-κB effects by ARRB/PP2A signaling^18^, PP2A inhibitor okadaic acid (OA) rescued the inhibitory effect of DA on NF-κB and M1 differentiation (Fig. S5A–D). Taken together, these data suggest that DA-DRD5 signaling specifically inhibits Lps/Ifn-γ-mediated NF-κB signaling and thereby inhibits M1 macrophage polarization.

Next, we use RNA-seq to analyze the transcriptional profiles of IL-4/IL-13-stimulated WT and *DRD5*^*−/−*^ M2 macrophage in the absence or presence of DA. PCA revealed that an entirely different clustered pattern in DA-treated M2 WT BMDMs compared with those of M2 WT, M2 *DRD5*^*−/−*^, and DA-stimulated M2 *DRD5*^*−/−*^ cells (Fig. 4E). Moreover, GSEA showed that CREB_PATHWAY was the top upregulated pathways in M2 WT BMDMs after DA treatment, and CREB_PATHWAY was also the top-ranking gene set in M2 WT BMDMs relative to *DRD5*^*−/−*^ cells in the presence of DA (Fig. 4F). Consistently, the immunoblotting analysis showed that DA significantly increased the phosphorylation of CREB in WT BMDMs after IL-4/IL-13 stimulation, but such effects of DA were severely impaired in *DRD5*^*−/−*^ BMDMs. Protein kinase A (PKA) is a well-known critical upstream protein kinase of CREB and can be activated by DA-DRD5 signaling^18^. Therefore, we next detected the phosphorylation level of PKA catalytic (PKAc) subunits, which phosphorylate the downstream targets such as CREB and ATF1^23,24^. The upregulation of phosphorylation of PKAc by DA was observed in WT but not *DRD5*^*−/−*^ BMDMs after stimulation with IL-4 and IL-13 (Fig. 4G). Moreover, the heatmap analysis also displayed a significant increase in the expression of a variety of genes associated with M2 macrophage and CREB signaling, such as *Arg1, Chil3, Mrc1, Socs1, Il10*, in WT but not *DRD5*^*−/−*^ BMDMs under M2 conditions in the presence of DA (Fig. 4H). Additionally, the inhibitor of CREB KG501 blocked the enhanced effects of DA/DRD5 on M2 differentiation (Fig. S5E–G). Collectively, these data clearly demonstrate that DA-DRD5 signaling can promote IL-4/IL-13-triggered M2 macrophage polarization through its activation of the CREB pathway.

### The deficiency of DRD5 signaling increased M1 macrophages but reduced M2 cells in the colon of DSS colitis mice

**Fig. 5:**
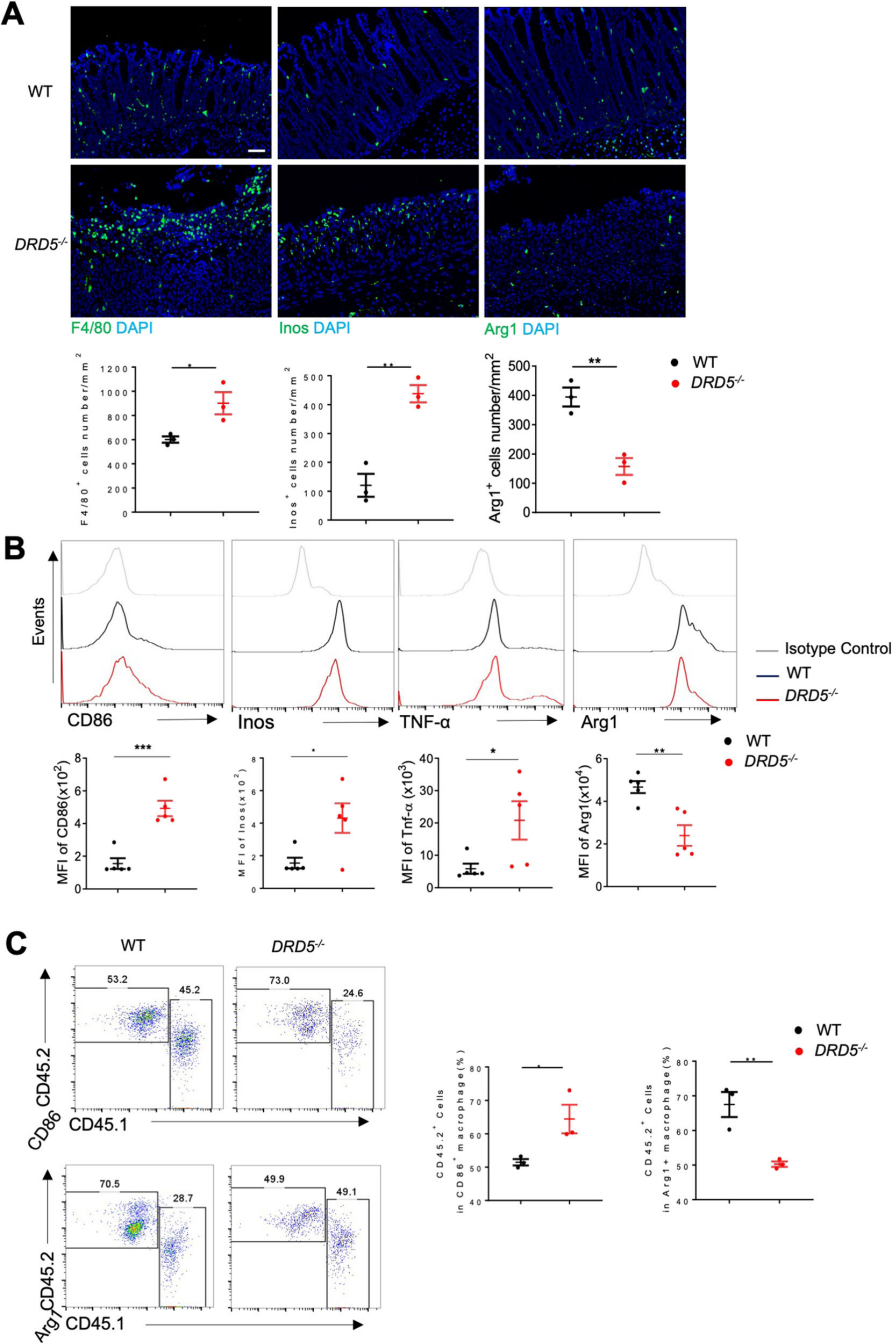
The deficiency of DRD5 signaling increased colonic M1 macrophages but reduced M2 cells in the colon of DSS colitis mice. **A** Immunofluorescent labeling of F4/80^+^, Arg1^+^, and Inos^+^ colonic macrophages of WT and *DRD5*^*−/−*^ mice on day 6 after DSS treatment (top). The cell numbers are quantified by Image pro plus (bottom) (*n* = 3). Scale bars, 200 μm. **B** Flow cytometry analysis of colonic macrophages of WT and *DRD5*^*−/−*^ mice (*n* = 5 mice per group) on day 6 after DSS treatment as indicated. Data are presented as representative plots (top) and summary graphs of quantified percentages (bottom). **C** Flow cytometry analysis of the CD45.2^+^ cells percentage in Arg1^+^ and CD86^+^ colonic macrophages of lethally irradiated mice reconstituted with *DRD5*^*−/−*^ CD45.2/WT CD45.1 bone marrow (1:1 ratio), and a control WT CD45.2/WT CD45.1 group (1:1 ratio) on day 6 after DSS treatment (*n* = 3 mice per group). Data are presented as representative plots (left) and summary graphs of quantified percentages (right). Data are pooled from two or three independent experiments. Error bars show means ± SEM. *p < 0.05, **p < 0.01, ***p < 0.001. Two-tailed unpaired student’s *t*-test (**A**, **B**, **C**).

To further investigated whether DA-DRD5 signaling could regulate colonic M1/M2 polarization in vivo to protect against colitis, we next characterized macrophages in the colon from untreated and DSS-induced colitis mice. An increase in the number of F4/80^+^ macrophages was observed in the colons of *DRD5*^*−/−*^ colitis mice relative to those of WT mice by immunofluorescence staining. Moreover, immunofluorescence showed that *DRD5*^*−/−*^ mice had significantly increased numbers of Inos^+^ M1 cells but markedly reduced numbers of Arg1^+^ M2 cells in the colon compared with WT mice (Fig. 5A). Consistently, FACS revealed that compared with WT mice, the expression of CD86, Inos, and TNF-α in colonic macrophages sorted from *DRD5*^*−/−*^ mice were modestly increased, but significantly increased on day 6 after DSS treatment. Moreover, the expression of Arg1 in colonic macrophages was markedly reduced in *DRD5*^*−/−*^ mice during colitis (Fig. 5B and Fig. S6). Additionally, we further validated the requirements for DRD5 signaling in macrophage polarization by constructing mixed bone marrow chimeras. The sublethally irradiated mice were reconstituted with *DRD5*^*−/−*^ CD45.2/WT CD45.1 bone marrow (1:1 ratio), and a control WT CD45.2/WT CD45.1 group (1:1 ratio). We found that mice that received DRD5 deficient bone marrow cells had higher M1 polarization and lower M2 polarization than WT donors after colitis (Fig. 5C). Collectively, these data suggest DRD5 signaling is indispensable for regulating the balance of colonic macrophage polarization and thereby its protective role in DSS-induced colitis.

### The administration of DRD5 agonist attenuates the colitogenic phenotype of mice

**Fig. 6:**
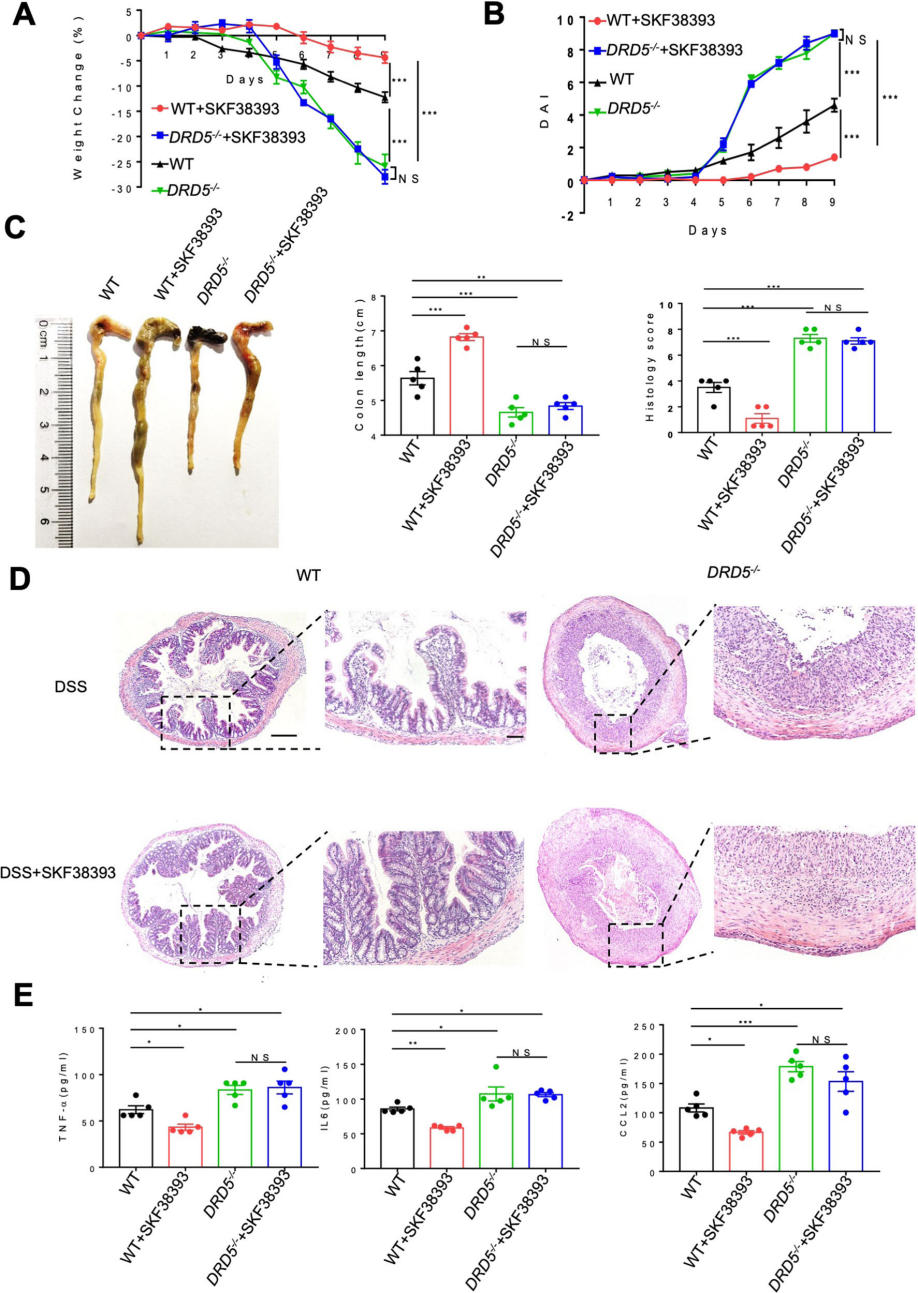
The administration of DRD5 agonist attenuates the colitogenic phenotype of mice. **A**, **B** Age-matched male WT and *DRD5*^*−/−*^ mice (*n* = 5 mice per group) were i.p. injected with D1-like agonist SKF-38393 at a dose of 10 mg/kg daily during DSS treatment. Weight changes (**A**) and disease activity index (DAI) (**B**) were monitored daily. **C** Gross morphology images of the DSS-induced colitis in WT mice or *DRD5*^*−/−*^ mice that were injected with DRD5 agonist, and colon length was measured on day 9. **D** Representative H&E-staining section of colons and histology score of mice in (**A**). Scale bars, 4×, 500 μm; 20×, 200 μm. **E** The concentration of TNF-α, IL-6, and CCL2 in the serum of WT and *DRD5*^*−/−*^ mice treated with SKF38393. Data are pooled from three independent experiments. Error bars show means ± SEM. *P < 0.05, **P < 0.01; ***P < 0.001; NS, not significant. multiple unpaired *t*-tests for (**A**, **B**) and One-way ANOVA with Sidak’s multiple comparisons test for (**C**–**E**).

Given that DRD5 signaling could protect against colitis, we were keen to assess if pharmacological activation of DRD5 could prevent colitis in mice. To test this, we treated WT and *DRD5*^*−/−*^ mice with intraperitoneal administration of the D1-like agonist SKF-38393 (10 mg/kg of body weight), which has a higher affinity to DRD5^25^. Such treatment significantly attenuated the clinical signs of colitis in WT mice as indicated by less weight loss, lower DAI score, less shortening in colon length, less histopathological findings, and lower serum cytokines of TNF-α, IL-6, and CCL2, whereas such effects of SKF-38393 were severely impaired in *DRD5*^*−/−*^ mice (Fig. 6A–E). Thus, these data further indicate a vital role of DRD5 signaling in the pathogenesis of colitis and suggest the development of potential therapeutic strategies to target DA-DRD5 signaling that might be useful for protection against colitis.

## Discussion

The most striking finding from this study is that DA-DRD5 signaling can inhibit the development of colitis by regulating the balance of colonic M1/M2 macrophages. Actually, DA or its agonists have been reported to act as protective agents in various rat ulcer models^26^. It is known that the gastrointestinal tract (GI) is one of the main sources of peripheral DA^27^. GI DA is mainly produced by the dopaminergic neuron in ENS and epithelial cells in the gut lumen^28,29^. There have been some studies that reported the reduction of dopamine content altered the status of immune cells in the intestine^30^. In this study, we further showed the role of DA via DRD5 in regulating the balance of colonic M1/M2 macrophage polarization and explained the underlying mechanism of DA in ameliorating colitis.

Macrophages in the GI tract represent the largest population of mononuclear macrophages in the body^31^. There is an increasing awareness of the role of macrophages in the regulation and development of gastrointestinal disease. The proinflammatory cytokines such as TNF-α released from M1 macrophages contribute to the development of IBD^32^. Besides, TNF-α neutralization induces CD206-positive M2 macrophages^33^ and contributes to mucosal healing^34^. IL-10, a potent cytokine derived from M2-like macrophages, is necessary for recovery from postoperative ileus and colitis^35,36^. DA receptors are widely distributed in almost all immune cell subsets including macrophages and serve to regulate the release of immune factors to affect immune cell function and inflammatory response^37^. In our study, we observed a high expression of DRD5 receptor in colonic LP macrophages, including F4/80^+^, Inos^+^M1, and Arg1^+^M2 cells. Consistent with the previous report that DA-DRD5 signaling suppresses macrophage-mediated inflammation^18^, we revealed that DA-DRD5 signaling is likely to play an important role in controlling the balance of colonic macrophage polarization, which is required for colonic homeostasis and prevent colitis. Notably, DA has recently been reported to inhibit LPS-induced NO production and inflammation in microglia, a type of central nervous system (CNS) macrophage, through DRD5-mediated signaling^38^, further suggesting an inhibitory role of DRD5 signaling in macrophage inflammation.

RNA-seq analysis revealed that DA-DRD5 signaling regulated macrophage polarization by inhibiting the NF-κB signaling pathway for the suppression of M1 macrophages while activating the CREB signal pathway to promote M2 macrophages. This finding is consistent with our previous report that dopamine uses DRD5 signaling to block TRAF6-mediated NF-κB inflammation^18^. Given that NF-κB signaling is critical for the induction of a large number of inflammatory M1 genes^39,40^, our study highlighted a vital role of DA-DRD5 signaling in suppressing NF-κB activation to control colonic M1 polarization. Additionally, PKA is a well-known critical upstream protein kinase of CREB and can be activated by DRD5 signaling^23,24^. Moreover, the PKA-CREB cascade has been reported to induce M2 macrophage-specific gene expression and M2 polarization^41–43^. Thus, our study also elucidates the mechanism map of DA-DRD5 signaling in facilitating M2 colonic macrophage polarization to control inflammation. Overall, in our model, colonic DA released by ENS dopaminergic neuron or colonic epithelial cells, acting to DRD5 receptor, inhibits M1 but promotes M2 macrophages polarization through the suppression of the NF-κB pathway and activation of the CREB pathway respectively, thereby driving anti-inflammatory protective effects in colitis (Fig. S7).

In this study, we found that the administration of D1-like agonist (prefer to target DRD5) attenuates the colitogenic phenotype of mice, suggesting targeting of DRD5 signaling could be a potential therapeutic manipulation to treat IBD. Moreover, the effects of DA on macrophages polarization might be not limited in IBD, because the dopaminergic signaling is widely distributed in the peripheral tissues of the body and the balance of M1/2 macrophage polarization has a close relationship with the development of various other diseases, such as infections^44^, lung injury^45^, and tumors^46^. Therefore, targeting the DA-DRD5 signaling pathway to regulate macrophage polarization has great potential to achieve a therapeutic effect to treat not only IBD but other macrophage-mediated inflammatory diseases.

## Materials and methods

### Mice

We used C57BL/6 background male mice in this study. *DRD5*^*−/−*^ mice were generated by Cyagen Biosciences Inc (Guangzhou, China) using CRISPR-Cas9 technology, as described previously^18^. To mark lamina propria macrophages, Rosa26-tdTomato mice were crossed with Cx3cr1-Cre mice. Rosa26-tdTomato and Cx3cr1-Cre mice were kindly provided by Dr. Jiawei Zhou (Institute of neuroscience, Chinese academy of sciences). For cohousing experiments, age-matched heterozygous male wild-type and *DRD5*^*−/−*^ mice from the same littermates were cohoused at a 1:1 ratio for 6 weeks. All mice were kept in a barrier facility, and all animal experiments were conducted following the procedure approved by the Ethical Review Committee for Laboratory Animal Welfare of Nanjing Medical University.

### Antibodies and reagents

Antibody to Inos (ab15323) was from Abcam. Anti-β-actin (A1978) antibody was from Sigma. Anti-Arg1 (PA585267), anti-F4/80 (14-4801-82), anti-PE IgG (35-4914-81), anti-PEcy7 IgG 25-4914-82 antibodies were from ebioscience. The anti-phosphorylated PKA C (5661) antibody was from SCT. Anti-PKA C-α (55388-1-AP) and anti-Drd5 (20310-1-AP/ADR-005) antibodies were from Proteintech/Alomone. Anti-phosphorylated IKKα/β (2697), anti-IKKβ (2370), anti-phosphorylated IκBα (9246) and anti-phosphorylated CREB (9198s) were from Cell Signaling Technology. Anti-CREB (48601-2) antibody was from SAB. IRDye 680RDanti-mouse (926-68070) and IRDye 800CW anti-rabbit (926-32211) were from LI-COR Biosciences. Anti-mouse-HRP and anti-rabbit-HRP were from Jackson ImmunoResearch. Flow antibodies including Anti-TCR-β-eFlour450 (48-5961-80), anti-CD45-AF700 (30-F11, 85-11-0112-81), anti-APC-CD45.1 (17-0453-82), anti-APC-eflour-780-CD45.2(47-0454-82), anti-CD11b-FITC (M1/70,85-12-0114-81), anti-F4/80-APC (BM8, 17-4801-82), anti-Ly6c-PE-Cy7 (HK1.4, 25-5932-82), anti-Ly6g-percpcy5.5 (48-9668-82), FVD-eFlour®506 (65-0866), anti-APC-TNF-α (17-7321-82) were from eBioscience. anti-CD11c (12-0114-82), anti-CD19 (17-0193-80) and anti-NK1.1-PE-cy7 (25-5941-81) were from Thermo. LPS (ALX-581-013-L002) was from EnzoLife Sciences. IL-4 (214-14), IL-13 (210-13-10), IFN-γ (500-M90) was from peprotech. SKF-38393 hydrobromide (0922) was from TOCRIS. KG501 (CSN22252) was from CSNpharm. OA (O7885-25UG) was from Sigma-Aldrich. DSS (DB001-38) was from TdB Consultancy.

### DSS-induced colitis

For acute experimental colitis induction, 6-8w age- and sex-matched WT or DRD5^*−/−*^ mice were distributed randomly according to their genotypes, which received 2.5% DSS from water for 6 days, followed by normal drinking water until the end of the experiment on days 9. During the experiment, body weights, stool, and body posture were monitored daily to assess the disease activity index (DAI) in a blinded fashion. The DAI is the combined score of weight loss compared to initial weight, stool consistency, and body posture. The scores are evaluated as follow: 0 (No weight loss or weight gain), 1 (5–10% weight loss), 2 (10–15% weight loss), 3 (>15% weight loss); stool consistency: 0 (normal and well-formed), 1 (very soft and formed), 2 (loose stool), 3 (bloody stools); body posture: 0 (Smooth fur without a hunchback), 1 (mild fur and hunchback), 2 (moderate fur and hunchback), 3 (Severe fur and heavy hunchback). Mice were sacrificed at the indicated time points, and colons were collected immediately for colon length measure, colonic macrophage analysis, and histology analysis.

### Bone marrow chimeras

The recipient mice were subjected to lethal-dose irradiation (10 Gy), and 1 d later, bone marrow cells (10 × 10^6^) derived from the tibiae and femurs of *DRD5*^*−/−*^ CD45.2/WT CD45.1 bone marrow (1:1 ratio), and a control WT CD45.2/WT CD45.1 group (1:1 ratio) were i.v. injected into lethally irradiated mice. After 8 weeks, the chimeric mice were then subjected to DSS induction.

### D1-like agonist SKF-38393 treatment in DSS-induced colitis mice

The D1-like agonist SKF-38393 was dissolved in water. The agonist was i.p. injected into WT and *DRD5*^*−/−*^ mice at a dose of 10 mg/kg daily, starting 1 d before the DSS challenge.

### Histological analysis

For histology, tissue sections were stained with hematoxylin & eosin (H&E). Histology was scored in a blinded fashion as a combination of inflammatory cell infiltration (score 0–4) and intestinal architecture damage (score 0–4). The presence of occasional inflammatory cells in the lamina propria was scored as 0; increased numbers of inflammatory cells in the lamina propria was scored as 1; inflammatory cells extending into the mucosa and submucosa was scored as 2; inflammatory cells extending into the mucosa, submucosa, and sometimes transmural infiltration was scored as 3; and severe transmural extension of the infiltrate was scored as 4. For architecture damage, no mucosal damage was scored as 0; focal erosions were scored as 1; slight crypt loss and focal ulcerations was scored as 2; extended ulcerations and moderate crypt loss was scored as 3; and extensive crypt loss, mucosal damage, and extension into deeper structures of the bowel wall was scored as 4. The total histologic score was derived by summing each individual score. Images were acquired with a Nikon 50i inverted microscope.

### M1/M2 polarization

For isolation of BMDMs, tibias and femurs were removed from WT or *DRD5*^*−/−*^ mice by sterile techniques, and the bone marrow was flushed with fresh medium. BMDMs were plated in DMEM supplemented with 10% FBS in the presence of 10% L929 conditioned medium for 4–6 d at 37 °C in a humidified atmosphere of 5% CO_2_. Primary BMDMs were seeded (1.5 × 10^6^ cells per well) in 12-well plates and were grown for 24 h. Cells were then stimulated with LPS (100 ng/ml), IFN-γ (50 ng/ml) or IL-4 (10 ng/ml), IL-13 (10 ng/ml) for 12 h to induce M1 or M2 macrophages differentiation with or without DA at different concentrations of 1, 10, 20, 50 μM. In addition, 10 μM MAO inhibitor of PLZ and COMT inhibitor of DNC were added to test the lowest effective concentration of DA.

### RT-qPCR

Total RNA was extracted by using TRIzol reagent (Life) and subjected to cDNA synthesis. Quantitative RT-PCR was performed using SYBR Green Supermix (Vazyme) according to the manufacturer’s instructions. The following primers were used:

**Table:** 

*Drd1*	S	AS
	5-CCGCTGTCATCAGGTTTCG-3	5-GATGTCAAAGGCTACCCAAATG-3
*Drd2*	S	AS
	5-CATCTCTTGCCCACTGCTCTT-3	5-CGATGGAGGAGTAGACCACGA-3
*Drd3*	S	AS
	5-CATCATCTTTGGCAACGGTCT-3	5-CGGCTGAAATTCCAGACTCC-3
*Drd4*	S	AS
	5-GGTGTGTTGGACGCCTTTCT-3	5-TTGAGGGCACTGTTGACATAGC-3
*Drd5*	S	AS
	5-CTATTTCCAGACCCTTCCGCT-3	5-CTGCCTTGTCTCTGTGCCAAT-3
*Inos*	S	AS
	5-AGGGAATCTTGGAGCGAGTTG-3	5-TGAGGGCTTGGCTGAGTGAG-3
*Il-6*	S	AS
	5-CTTGGGACTGATGCTGGTGAC-3	5-GCCATTGCACAACTCTTTTCTC-3
*Tnf*	S	AS
	5-TACTGAACTTCGGGGTGATCG-3	5-TCCTCCACTTGGTGGTTTGC-3
*Ym1*	S	AS
	5- CATTGGAGGATGGAAGTTTGG -3	5-GGTACTGCCAGTCCAGGTTGA-3
*Mrc1*	S	AS
	5-CTCAACCCAAGGGCTCTTCTAA -3	5-AAGGTGGCCTCTTGAGGTATGT-3
*Arg1*	S	AS
	5-GTCTGGCAGTTGGAAGCATCT -3	5-GCTGGTTGTCAGGGGAGTGT-3
*Hprt*	S	AS
	5-GTCCCAGCGTCGTGATTAGC-3	5-TGGCCTCCCATCTCCTTCA-3
*Gapdh*	S	AS
	5-TGGATTTGGACGCATTGGTC-3	5-TTTGCACTGGTACGTGTTGAT-3

### ELISA

Primary BMDMs were seeded (1.5 × 10^6^ cells per well) in 12-well plates and were grown for 24 h. Cells were then untreated or treated DA and stimulated with LPS (100 ng/ml), IFN-γ (50 ng/ml) or IL-4 (10 ng /ml), IL-13 (10 ng/ml) for 12 h. Conditioned media from these groups above and the serum from DSS or SKF38393 treated mice were collected and measured for levels of IL-6 (DY406), TNF-α (DY410) and CCL2 (DY479) according to manufacturer’s instructions (R&D Systems).

### Immunoblotting

Primary BMDMs were seeded (1.5 × 10^6^ cells per well) in 12-well plates and were grown for 24 h. Cells were then untreated or treated DA and stimulated with LPS (100 ng/ml), IFN-γ (50 ng/ml) or IL-4 (10 ng /ml), IL-13 (10 ng/ml). BMDMs were lysed in SDS buffer and boiled for 10 min. Samples were resolved by SDS-PAGE, transferred to nitrocellulose membranes, and analyzed by immunoblot with the appropriate antibodies.

### Immunofluorescence staining

Tissue sections were incubated with primary antibody to Drd5, F4/80, Inos, and Arg1 sections at 4 °C overnight, and then incubated with secondary antibody as indicated. The nuclei were counterstained with 4,6-diamidino-2-phenylindole (DAPI) (sigma). Slides were dried and mounted using ProLong Antifade mounting medium (Beyotime Biotechnology). At last, slides were visualized using a Nikon 50i fluorescent microscope. The number of macrophages from three images that were randomly selected from each tissue section were quantified using Image pro plus. The cell number shown in Fig. 6A (*y*) is derived from this formula. y = N/S (*y*, number of cells per mm^2^; N, the total number of cells per tissue section; S, area).

### FACS analysis and sorting of colonic epithelial and immune cells

Colons were excised and washed thoroughly by flushing with PBS several times. They were opened longitudinally and transferred into PBS contained 1 mM DTT and 5 mM EDTA and shaken for 20 min at 37 °C, repeated twice. All supernatants were collected and passed through a 70 μm cell strainer for staining, then the epithelial cells (CD45^-^CD326^+^) were sorted out by flow cytometry. The remaining tissues were digested with DMEM media contained 1 mg/ml collagenase IV(Sigma) and 10 U/ml DNAse I (Roche) for 25 min. After repeating twice, all supernatants were collected and passed through a 70 μm cell strainer for staining. The immune cells were collected from the middle layer of the liquid surface after density gradient centrifugation with 40/80% Percoll (GE Healthcare). After intensive washing, single suspensions were stained with FVD eFlour® 506, anti-CD45, anti-CD45.1, anti-CD45.2, anti-CD11b, anti-F4/80, anti-CD86, anti-CD206, anti-Inos, anti-Arg1, anti-TNF-α for FACS analysis. All flow cytometry was performed on an Attune NxT flow cytometer (Thermo Fisher) and data were analyzed by FlowJo 10 software. For colonic immune cells sorting, we use flow cytometry to sort out CD45^+^ immune cells (CD45^+^CD326^-^), T cells (CD45^+^TCR-β^+^), B cells (CD45^+^CD19^+^), NK cells (CD45^+^NK1.1^+^), macrophages (CD45^+^F4/80^+^CD11b^+^), monocytes (CD45^+^Ly6c^+^CD11b^+^), DC cells (CD45^+^CD11c^+^), neutrophils (CD45^+^Ly6G^+^), intrinsic lymphoid cells (CD45^+^Lin^-^).

### RNA-Seq analysis

BMDMs from WT or *DRD5*^*−/−*^ mice were untreated or treated DA and stimulated with LPS (100 ng/ml), IFN-γ (50 ng/ml) or IL-4 (10 ng /ml), IL-13 (10 ng/ml) and then collected for RNA extraction. RNA samples were constructed and sequenced on a BGISEQ-500 (Beijing Genomic Institution, BGI). The filtered data were mapped to the mouse genome (GRCm38.p5) through HISAT2. For gene expression analysis, the matched reads were calculated and then normalized to FPKM. Fold changes were calculated for all possible comparisons and a 1.2-fold and FDR < 0.001 cutoff was used to select genes with expression changes for the Heat map. The whole-genome were analyzed for the Principle component (PC). GSEA analysis was performed using the R package, using whole-genome as target genes. The Raw data files and processed files have been uploaded to Gene Expression Omnibus public database (Accession: GSE159206).

### 16S ribosomal RNA gene sequencing

Microbial DNA was extracted from fecal samples of the indicated mice using the TIANamp Stool DNA Kit (TIANGEN) according to the manufacturer’s protocols. The final DNA concentration and purification were determined by Onedrop, and the quality of DNA was determined by agarose gel electrophoresis. The purified DNA amplicons were then added with Illumina adapters by ligation (TruSeq DNA LT Sample Prep Kit), the adapter-ligated DNA fragments were further pooled in equimolar and paired-end sequenced (2 × 300) on an Illumina MiSeq platform for sequencing according to the standard protocols by Majorbio Bio-Pharm Technology Co. Ltd. (Shanghai, China). Operational taxonomic units (OTUs) were clustered with 97% similarity cutoff using UPARSE (version 7.1 http://drive5.com/uparse/) with a novel ‘greedy’ algorithm that performs chimera filtering and OTU clustering simultaneously. The taxonomy of each 16S rRNA gene sequence was analyzed by the RDP Classifier algorithm (http://rdp.cme.msu.edu/) against the Silva (SSU123) 16S rRNA database using a confidence threshold of 70%. The raw reads were deposited into the NCBI Sequence Read Archive (SRA) database (SRA accession: PRJNA664271).

### HPLC analysis

HPLC analysis was employed to measure dopamine from the distal colon. This analysis used a DIONEX HPLC system with a Coulochem III Electrochemical Detector together with a Uniget C-18 reverse phase microbore column as the stationary phase. The mobile phase consisted of buffer [1.7 mM OSA, 0.05 mM Na-EDTA, 90 mM NaH_2_PO_4_·2H_2_O, and 50 mM C_6_H_8_O_7_·H_2_O] and acetonitrile. The flow rate was 0.2 ml/min, and the working electrode was set at 350 mV versus Ag/Ag/Cl reference electrode. Detection gain was 100 nA and filter was 5 s.10 mL of the sample supernatant was directly injected into the HPLC for analysis. Standard dopamine (Sigma) was used to quantify and identify the peaks on the chromatographs. The detection limits for dopamine was determined by analyzing the known concentrations of dopamine in the HPLC system under the set condition. For this purpose, standard solutions of 1 mg per ml were made with pure dopamine and diluted accordingly to the desired concentrations of the stock solutions for running on HPLC. Concentration of dopamine was determined using the following formula: y(nmol/L) = 0.0217x–0.0132 (r2 = 0.9998) (y, peak area; x, analyte concentration in μM). To more effectively compare dopamine concentrations between studies, all values of tissues were converted to final molar concentrations by dividing original tissue weights and multiplying the density of tissues which we averaged to be around 1 kg/L.

### Statistical analyses

The data were analyzed by GraphPadPrism 7.0 and GraphPadPrism 8.0 software and are presented as the mean ± standard error of the mean (SEM). The statistics were analyzed by using a two-tailed unpaired *t*-test for two groups, one-way ANOVA for multiple groups. *P* values were provided as ∗*p* < 0.05, ∗∗*p* < 0.01 and ∗∗∗*p* < 0.001.

## Supplementary information

Supplementary Figures

## Data Availability

Sequencing data are deposited into the Gene Expression Omnibus (accession no. SRP574780 and GSE159206).
