# Supplementary material for: DA-DRD5 signaling controls colitis by regulating colonic M1/M2 macrophage polarization
Source: Cell Death Dis. 2021 May 17;12(6):500. doi: 10.1038/s41419-021-03778-6 (PMC8129081; doi:10.1038/s41419-021-03778-6)
Supplement: Supplementary file 1 — Supplementary Figures [file 41419_2021_3778_MOESM1_ESM.pdf]

Supplementary Fig 1

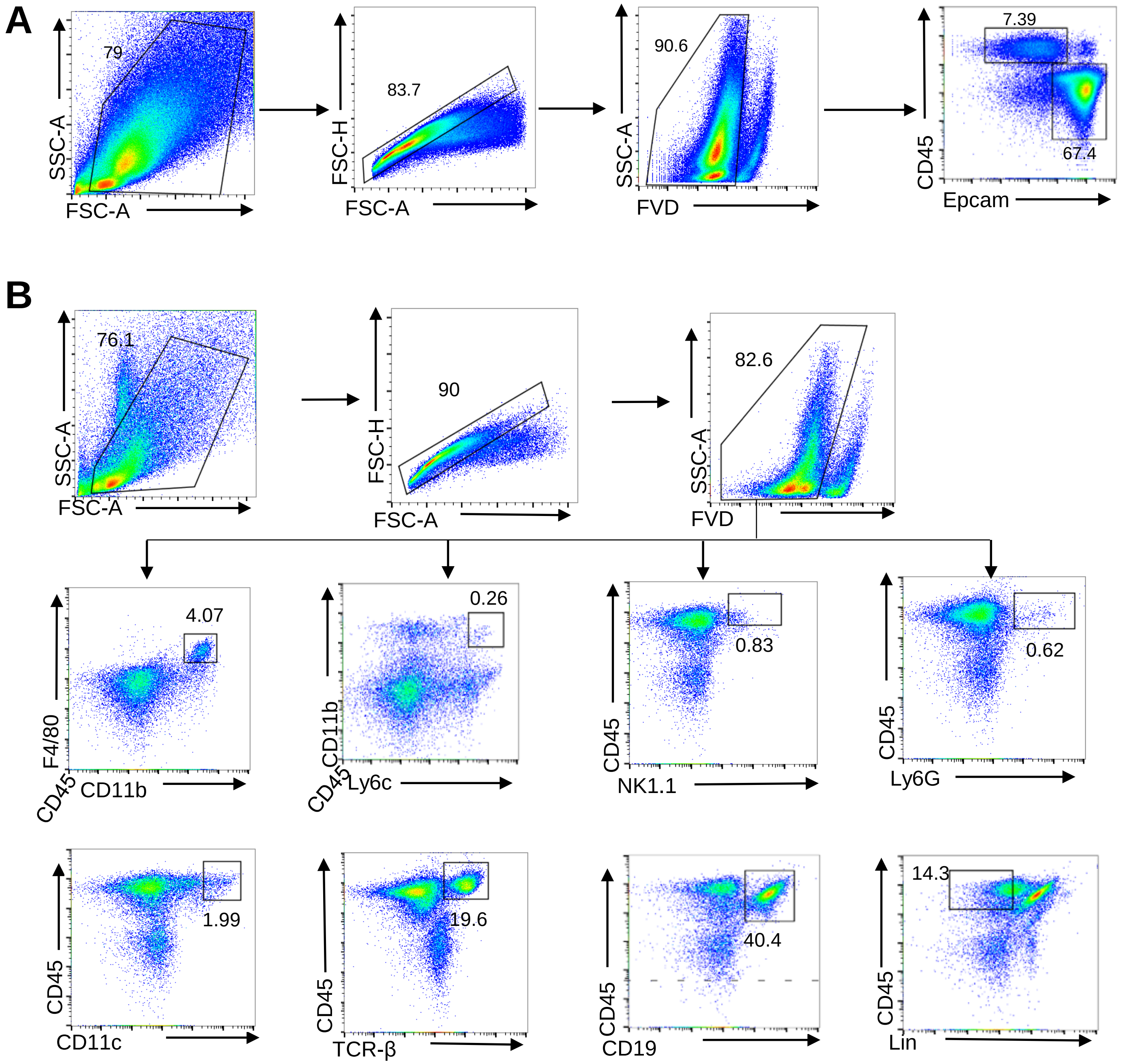

Supplementary Figure 1. FACS sorting strategy of colonic epithelial and immune cells.

(A) Representative flow cytometry profiles from colonic epithelial and immune cells in WT mice for gating strategy and FACS-sorting.

(B) Representative flow cytometry profiles from colonic immune cells in WT mice for gating strategy and FACS-sorting.

Supplementary Fig 2

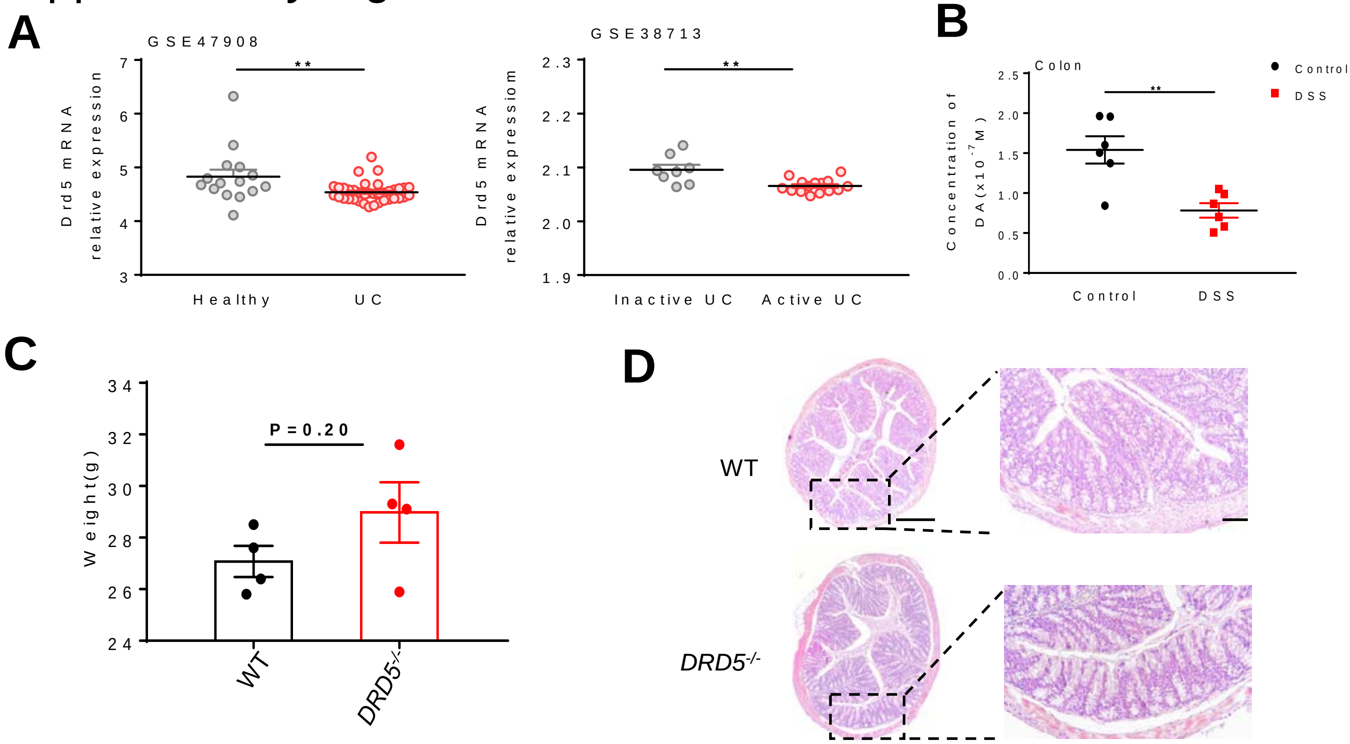

Supplementary Figure 2. Colonic DA levels after DSS colitis, and the weight and colonic histopathology in untreated WT and *DRD5*$^{-/-}$ mice.

(A) The analysis of *Drd5* mRNA levels in the colon of healthy individuals and UC patients using GEO data sets GSE47908 and GSE38713.

(B) HPLC analysis of dopamine levels in the colon from wild-type mice with or without DSS treatment.

(C) Weight of WT and *DRD5*$^{-/-}$ mice (n=4 mice per group) were monitored.

(D) Representative H&E-stained section colons of WT and *DRD5*$^{-/-}$ mice. Scale bars, 4X, 500μm; 20X, 200μm.

Supplementary Fig 3

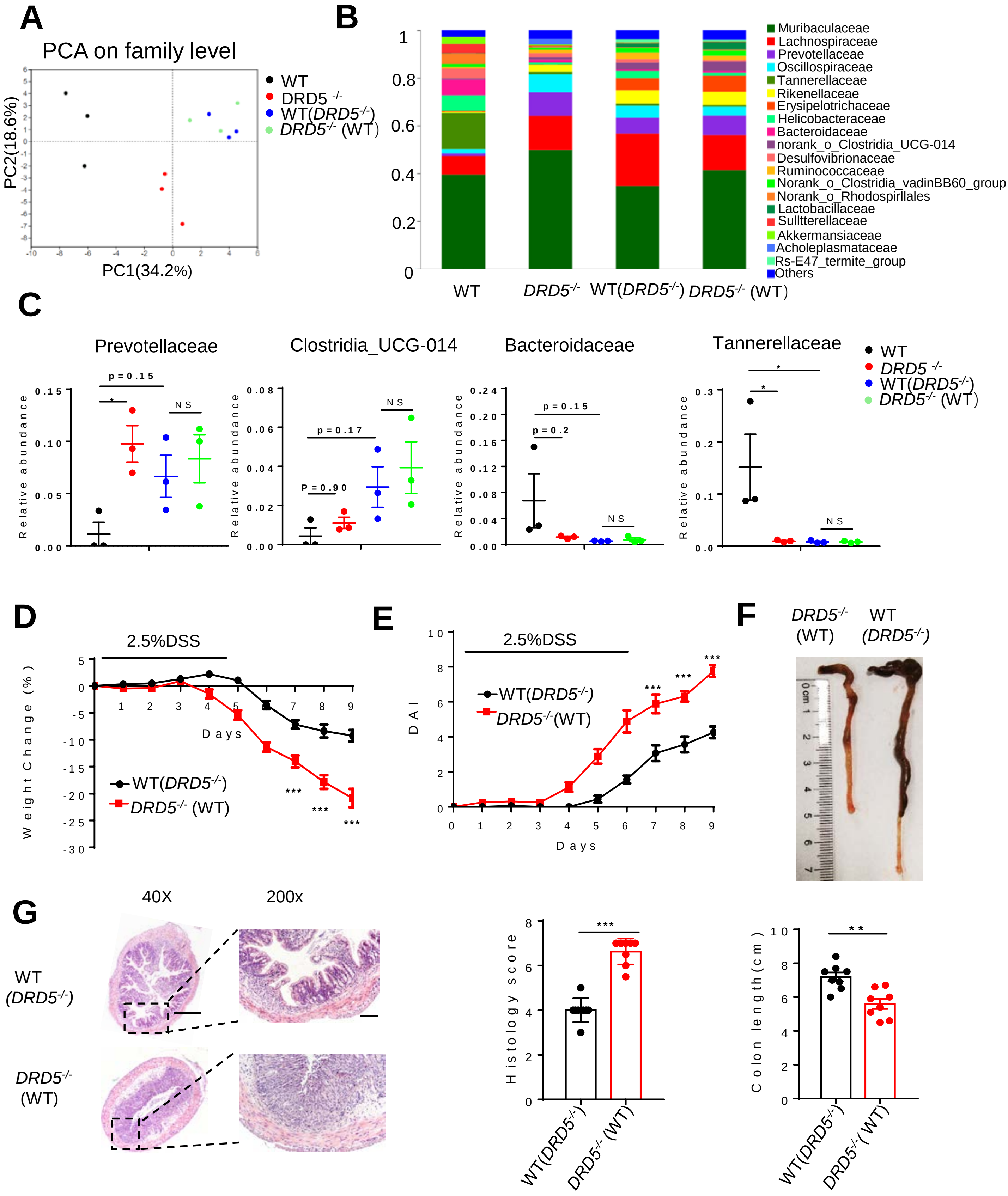

Supplementary Figure 3. 16S rRNA-seq analysis of fecal microbiota and histopathological analysis from single-housed and cohoused WT and *DRD5*$^{-/-}$ mice.

(A) Principal component (PC) analysis of clustered microbiota in **s**ingle-housed and cohoused WT and *DRD5*$^{-/-}$ mice (n=3).

(B) The percent of community abundance on family level in **s**ingle-housed and cohoused WT and *DRD5*$^{-/-}$ mice.

(C) The relative abundance of Prevotellaceae, Clostridia_UCG-014, Bacteroidaceae, and Tannerellaceae in **s**ingle-housed and cohoused WT and *DRD5*$^{-/-}$ mice.

(D, E) Littermate male WT and *DRD5*$^{-/-}$ mice (n=8 mice per group) were cohoused for six weeks and then were given 2.5% DSS in their drinking water for six days and distilled water for three additional days before sacrifice. Weight (D) and DAI (E) of cohoused WT and *DRD5*$^{-/-}$ mice were monitored daily.

(F) Gross morphology images of colons and colon lengths of cohoused WT and *DRD5*$^{-/-}$ mice on day 9 after DSS treatment.

(G) Representative H&E-stained section colons and histology scores of cohoused WT and *DRD5*$^{-/-}$ mice sampled on day 9 after DSS treatment. Scale bars, 4X, 500μm; 20X, 200μm.

Data are pooled from three independent experiments. Error bars show means ± SEM. *$p < 0.05$,**, $P<0.01$; ***, $P<0.001$. two-tailed unpaired student’s t-test for C, F, G. multiple unpaired t-tests for D, E.

Supplementary Fig 4

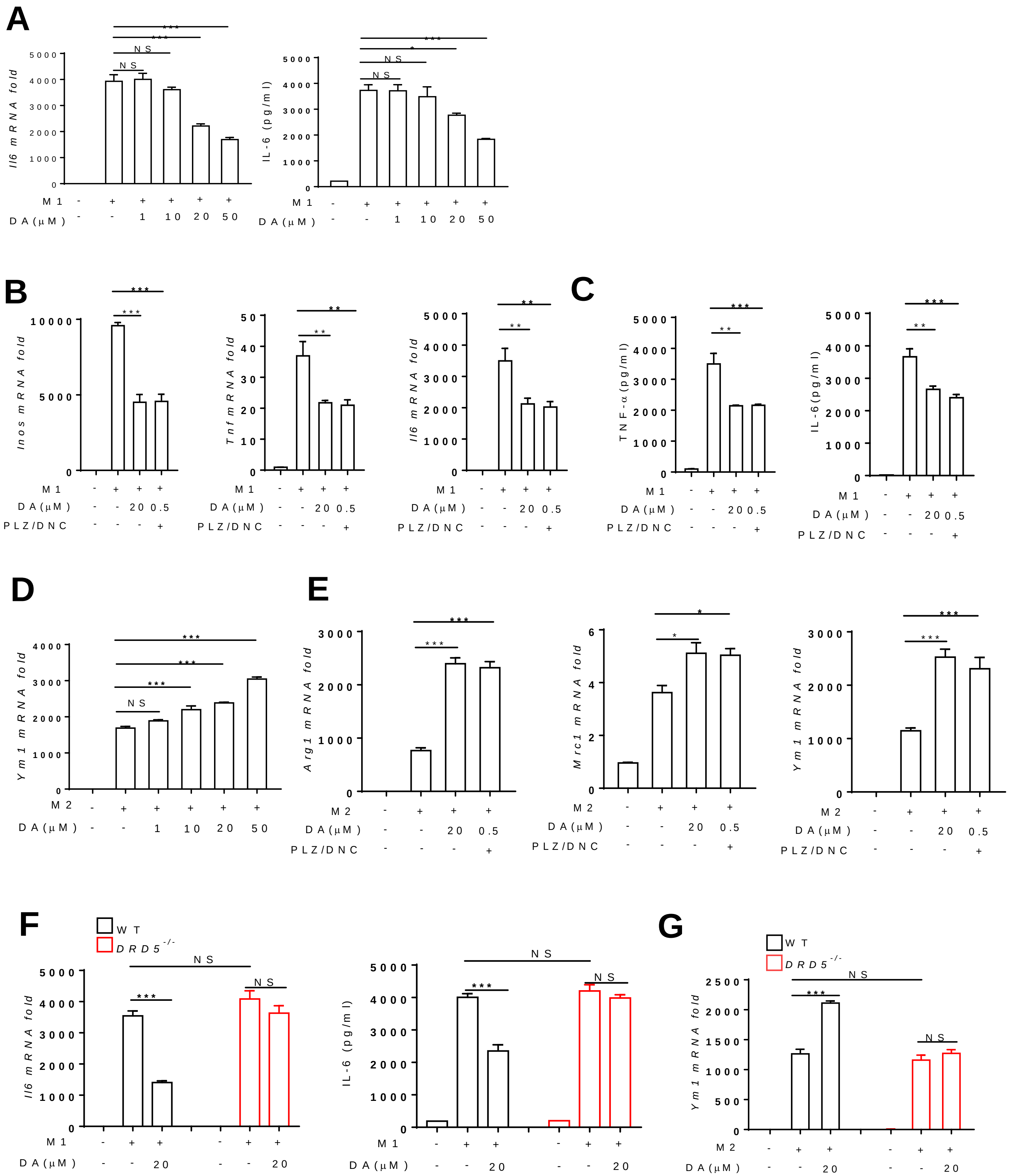

Supplementary Figure 4. The deficiency of DA-DRD5 signaling inhibits M1 but enhances M2 macrophage polarization in vitro.

(A) RT-qPCR analysis of *IL-6* mRNA expression in WT BMDMs treated with various doses of DA and stimulated with LPS/IFN-γ (M1) for 12h (left). ELISA analysis of IL-6 in supernatants from WT BMDMs treated with various doses of DA and stimulated with LPS/IFN-γ (M1) for 12h (right).

(B) RT-qPCR analysis of *Inos*, *Tnf* and *Il6* mRNA expression in BMDMs treated with phenelzine (PLZ, 10 μM) plus 3,5-dinitrocatechol (DNC, 10 μM) and different doses of DA and stimulated with LPS/IFN-γ (M1) for 12h.

(C) ELISA analysis of TNF-α and IL-6 in supernatants from WT BMDMs treated with phenelzine (PLZ, 10 μM) plus 3,5-dinitrocatechol (DNC,10 μM) and different doses of DA and stimulated with LPS/IFN-γ (M1) for 12h.

(D) RT-qPCR analysis of *Ym1* mRNA expression in WT BMDMs treated with various doses of DA and stimulated with IL-4/IL-13 (M2) for 12h.

(E) RT-qPCR analysis of *Arg1*, *Mrc1*and *Ym1* mRNA expression in BMDMs treated with phenelzine (PLZ, 10 μM) plus 3,5-dinitrocatechol (DNC, 10 μM) and different doses of DA and stimulated with IL-4/IL-13 (M2) for 12h.

(F) RT-qPCR analysis of *IL-6* mRNA expression in WT and *DRD5*$^{-/-}$ BMDMs treated with DA (20 μM) and stimulated with LPS/IFN-γ (M1) for 12h (left). ELISA analysis of IL-6 in supernatants from WT and *DRD5*$^{-/-}$ BMDMs treated with DA (20 μM) and stimulated with LPS/IFN-γ (M1) for 12h (right).

(G) RT-qPCR analysis of *Ym1* mRNA expression in WT and *DRD5*$^{-/-}$ BMDMs treated with DA (20 μM) and stimulated with IL-4/IL-13 (M2) for 12h.

Data are pooled from three independent experiments. Error bars show means ± SEM. *$p < 0.05$, **, $P<0.01$; ***$p < 0.001$; NS, not significant. One-way ANOVA with Sidak's multiple comparisons test for A-G.

Supplementary Fig 5

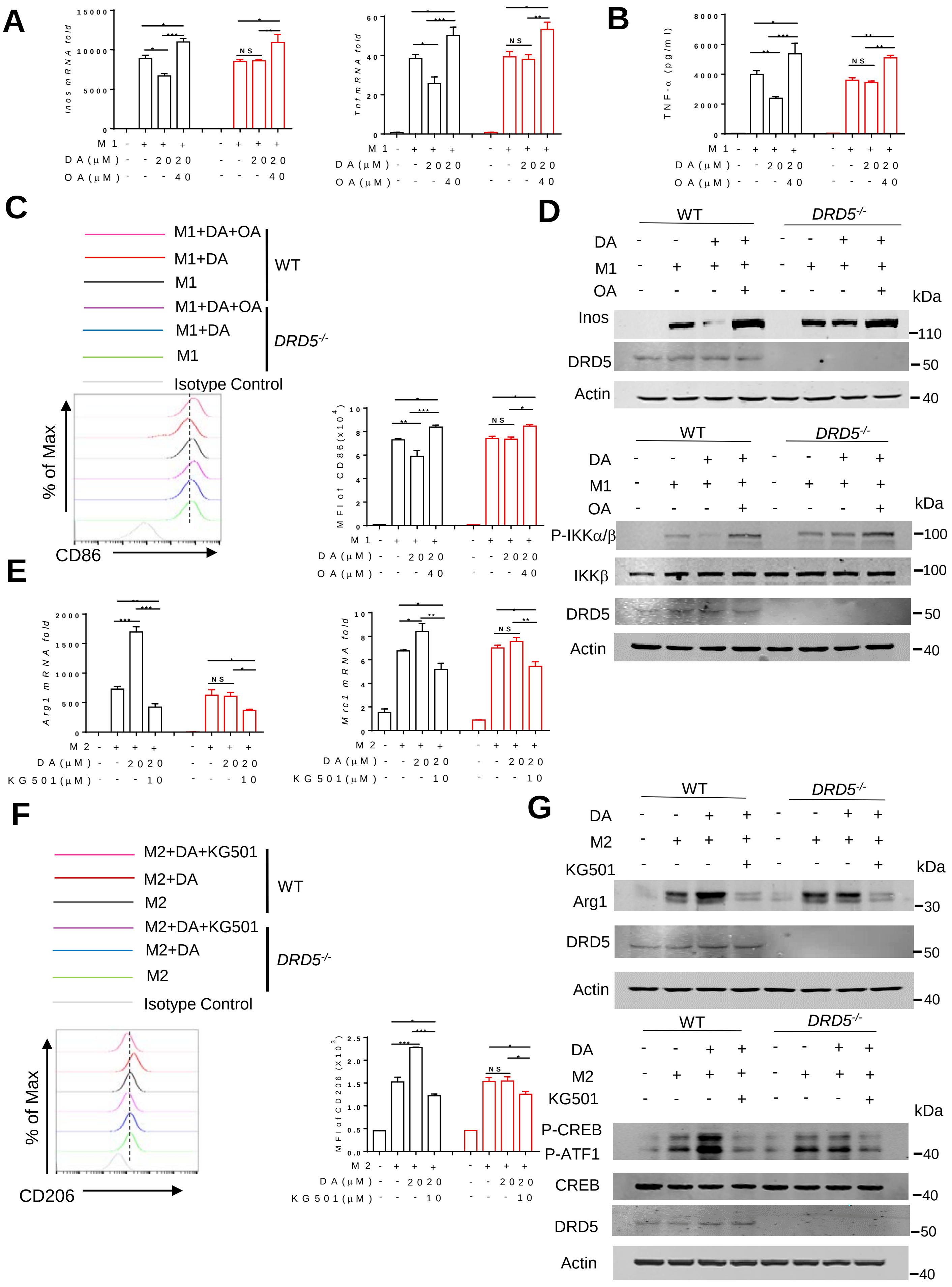

Supplementary Figure 5. DA-DRD5 signaling inhibits M1 polarization by negatively regulating NF-κB signaling, but enhances M2 macrophage differentiation through the CREB pathway.

(A) RT-qPCR analysis of *Inos* and *TNF-α* mRNA expression in WT and DRD5$^{-/-}$ BMDMs treated with DA (20 μM) and OA (Okadaic Acid, 40 μM) and stimulated with LPS/IFN-γ (M1) for 12h.

(B) ELISA analysis of TNF-α in supernatants from WT and *DRD5*$^{-/-}$ BMDMs treated with DA and OA and stimulated with LPS/IFN-γ (M1) for 12h.

(C) Flow cytometry analysis of M1 marker CD86 of WT and *DRD5*$^{-/-}$ BMDMs treated with DA and OA and stimulated with LPS/IFN-γ (M1) for 12h.

(D) Immunoblot analysis of Inos, (p-), total IKKα/β, DRD5 and Actin in lysates of WT and *DRD5*$^{-/-}$ BMDMs treated with DA and OA and stimulated with LPS/IFN-γ (M1) for 12h or 0.5h respectively.

(E) RT-qPCR analysis of *Inos* and *Arg1* and *Mrc1* mRNA expression in WT and *DRD5*$^{-/-}$ BMDMs treated with DA (20 μM) and KG501 (2-naphthol-AS-E-phosphate, 10 μM) and stimulated with IL-4/IL-13 (M2) for 12h.

(F) Flow cytometry analysis of M2 marker CD206 of WT and *DRD5*$^{-/-}$ BMDMs treated with DA and KG501 and stimulated with IL-4/IL-13 (M2) for 12h.

(G) Immunoblot analysis of Arg1, (p-), total CREB, DRD5 and Actin in lysates of WT and *DRD5*$^{-/-}$ BMDMs treated with DA and OA and stimulated with IL-4/IL-13 (M2), the time point of data collection for Arg1 and p-CREB is 12h or 0.5h respectively.

Data are pooled from three independent experiments. Error bars show means ± SEM. *, $P<0.05$; **, $P<0.01$; ***, $P<0.001$. two-tailed unpaired student's t-test for A-C, E-F.

Supplementary Fig 6

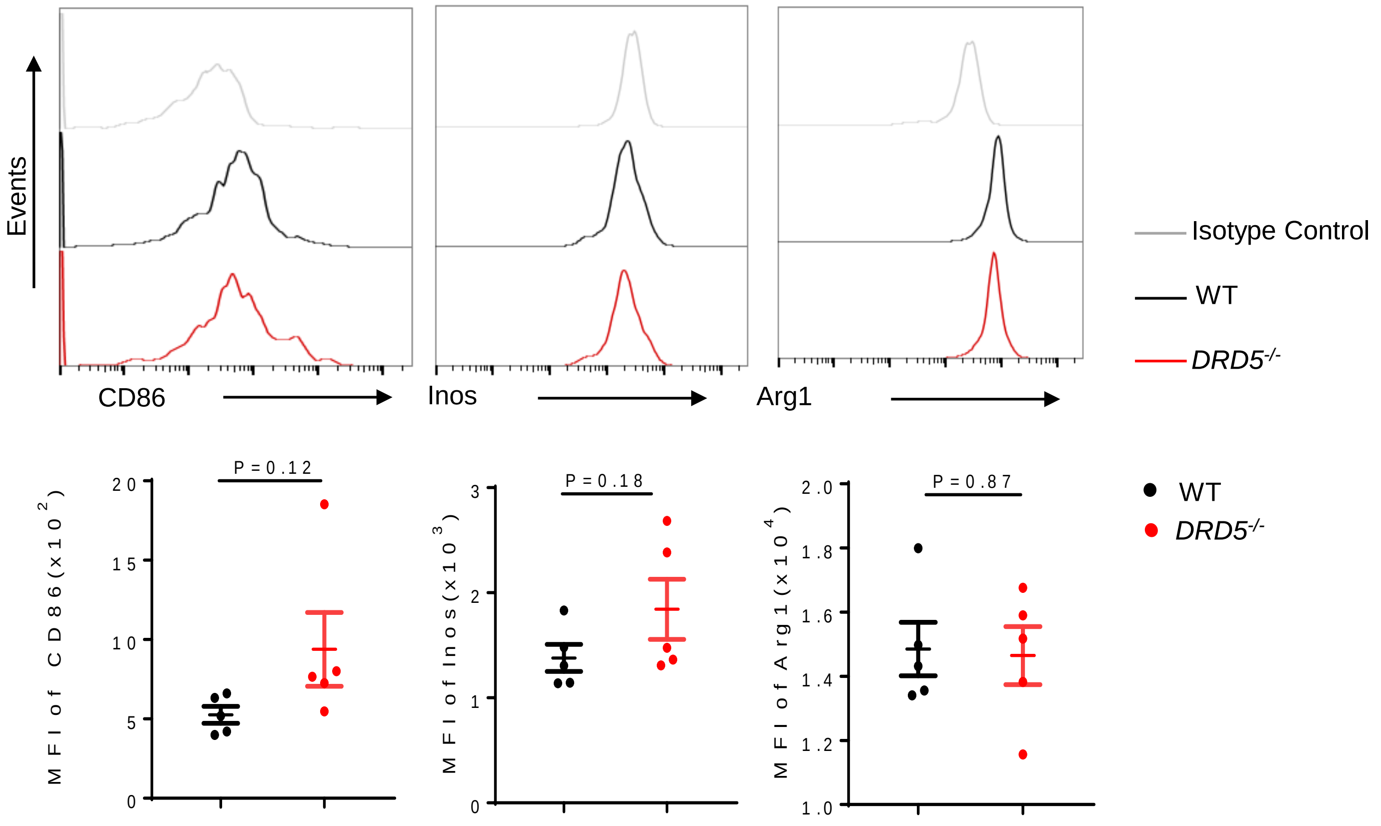

Supplementary Figure 6. The deficiency of DRD5 signaling increased colonic M1 macrophages but reduced M2 cells in the colon of mice.

Flow cytometry analysis of colonic macrophage of WT and *DRD5*$^{-/-}$ mice as indicated (n= 5 mice per group). Data are presented as representative plots (top) and summary graphs of quantified percentages (bottom). Data are pooled from three independent experiments. Error bars show means ± SEM. Two-tailed unpaired student's t-test for the figure.

Supplementary Fig 7

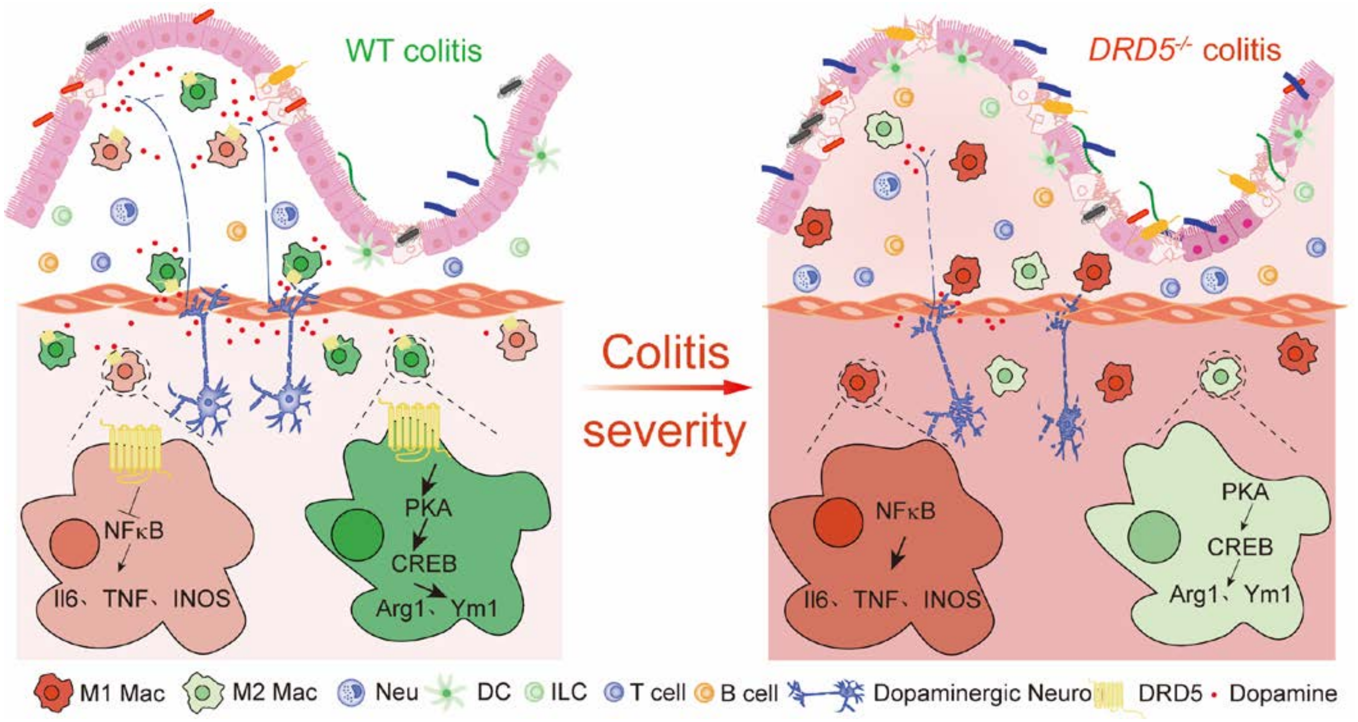

Supplementary Figure 7. Model for DA-DRD5 signaling function in controlling colitis by regulating the balance of M1/M2 macrophage polarization.

GI DA via DRD5 inhibits M1 but promotes M2 macrophage polarization through the suppression of the NF-κB pathway and activation of the CREB pathway respectively, thereby driving anti-inflammatory protective effects in colitis.
